# Automated quality control of T1-weighted brain MRI scans for clinical research datasets: methods comparison and design of a quality prediction classifier

**DOI:** 10.1162/IMAG.a.4

**Published:** 2025-05-28

**Authors:** Gaurav Bhalerao, Grace Gillis, Mohamed Dembele, Sana Suri, Klaus Ebmeier, Johannes Klein, Michele Hu, Clare Mackay, Ludovica Griffanti

**Affiliations:** Oxford Centre for Human Brain Activity, Wellcome Centre for Integrative Neuroimaging, Department of Psychiatry, University of Oxford, Oxford, United Kingdom; Department of Psychiatry, University of Oxford, Oxford, United Kingdom; Wellcome Centre for Integrative Neuroimaging, FMRIB, Nuffield Department of Clinical Neurosciences, University of Oxford, Oxford, United Kingdom; Nuffield Department of Clinical Neurosciences, University of Oxford, Oxford, United Kingdom

**Keywords:** brain MRI, classifier, DPUK, multisite, prediction, T1w, quality control

## Abstract

T1-weighted (T1w) MRI is widely used in clinical neuroimaging for studying brain structure and its changes, including those related to neurodegenerative diseases, and as anatomical reference for analysing other modalities. Ensuring high-quality T1w scans is vital as image quality affects reliability of outcome measures. However, visual inspection can be subjective and time consuming, especially with large datasets. The effectiveness of automated quality control (QC) tools for clinical cohorts remains uncertain. In this study, we used T1w scans from elderly participants within ageing and clinical populations to test the accuracy of existing QC tools with respect to visual QC and to establish a new quality prediction framework for clinical research use. Four datasets acquired from multiple scanners and sites were used (*N*= 2438, 11 sites, 39 scanner manufacturer models, 3 field strengths—1.5T, 3T, 2.9T, patients and controls, average age 71 ± 8 years). All structural T1w scans were processed with two standard automated QC pipelines (MRIQC and CAT12). The agreement of the accept–reject ratings was compared between the automated pipelines and with visual QC. We then designed a quality prediction framework that combines the QC measures from the existing automated tools and is trained on clinical research datasets. We tested the classifier performance using cross-validation on data from all sites together, also examining the performance across diagnostic groups. We then tested the generalisability of our approach when leaving one site out and explored how well our approach generalises to data from a different scanner manufacturer and/or field strength from those used for training, as well as on an unseen new dataset of healthy young participants with movement-related artefacts. Our results show significant agreement between automated QC tools and visual QC (Kappa = 0.30 with MRIQC predictions; Kappa = 0.28 with CAT12’s rating) when considering the entire dataset, but the agreement was highly variable across datasets. Our proposed robust undersampling boost (RUS) classifier achieved 87.7% balanced accuracy on the test data combined from different sites (with 86.6% and 88.3% balanced accuracy on scans from patients and controls, respectively). This classifier was also found to be generalisable on different combinations of training and test datasets (average balanced accuracy of leave-one-site-out = 78.2%; exploratory models on field strengths and manufacturers = 77.7%; movement-related artefact dataset when including 1% scans in the training = 88.5%). While existing QC tools may not be robustly applicable to datasets comprising older adults, they produce quality metrics that can be leveraged to train more robust quality control classifiers for ageing and clinical cohorts.

## 1 Introduction

Large-scale brain MRI datasets hold immense value for well-powered statistical analyses and cross-cohort investigations (Madan, 2022). The emergence of open science initiatives and platforms for sharing data has made it possible to combine data from multiple sites and studies (Markiewicz et al., 2021;Wilkinson et al., 2016). With the emergence of comprehensive neuroimaging pipelines (e.g., UK Biobank, Human Connectome Project, etc.), it is now feasible to obtain imaging-derived outcome measures on other datasets, including clinical populations (Littlejohns et al., 2020;Van Essen et al., 2013). In the ageing and dementia space, there is a wealth of clinical research datasets, made available through initiatives such as the Alzheimer’s Disease Neuroimaging Initiative (ADNI) and Dementias Platform UK (DPUK) (Bauermeister et al., 2020;Petersen et al., 2010). The aggregation of neuroimaging data obtained from clinical populations not only increases sample sizes but also facilitates the generation of reproducible and generalisable outcome measures, thus paving the way for innovative approaches in detecting brain biomarkers (Khanna et al., 2018;Van Horn & Toga, 2009). A substantial focus of neuroimaging research revolves around enhancing automated pipelines to produce reliable and relevant outcome measures from extensive datasets (Esteban et al., 2019;Frazier-Logue et al., 2022;Notter et al., 2023;Sherif et al., 2014). However, analysing large-scale datasets requires robust automated pipelines to ensure the generation of consistent measures across varied datasets. Despite the benefits, dealing with clinical research datasets poses an additional challenge in big data analysis due to higher heterogeneity, motion artefacts, and disease-related factors such as atrophy or other abnormalities (Andre et al., 2015;Nárai et al., 2022). As a result, it becomes crucial to identify and exclude scans of poor or unusable quality to ensure that the outcome measures generated by the automated processing pipelines are reliable.

While the MRI protocol may vary across datasets, a core component is a structural T1-weighted (T1w) scan. T1w MRI is used to examine brain structures, assess brain volume changes, and detect abnormalities, for example, those associated with neurodegenerative diseases. It is also used as anatomical reference for the analysis of other structural and functional imaging modalities, as it provides detailed anatomical information. The initial and crucial step in brain imaging analysis involves assessing the quality of T1w MRI scans. The effectiveness of subsequent steps, such as multimodal registration and morphometry estimation, relies heavily on the quality of these scans. Traditionally, researchers visually inspect scans before analysis, but this practice is not always feasible when dealing with large datasets. Removing too many scans after quality assessment can decrease the sample size, while including poor-quality scans can introduce biases into the resulting outcomes (Gilmore et al., 2021).

Several manual and (semi-) automated approaches have been developed for quality control (QC) on raw and processed T1w brain MRI scans (Hendriks et al., 2023;Klapwijk et al., 2019;Monereo-Sánchez et al., 2021;Raamana et al., 2023). Various rule-based QC approaches have been proposed considering the image background to assess scan quality, for example, using measures such as distortion (Woodard & Carley-Spencer, 2006), noise, and ghosting artefacts (Gedamu et al., 2008), derived from image background (Mortamet et al., 2009). Other rule-based QC approaches considered the image foreground to assess quality of the scans (Jang et al., 2018;Osadebey et al., 2018). Several automated machine learning approaches have been proposed, which extract quality measures from the images and are trained using visual QC labels to predict scan quality (pass or fail). For example,Alfaro-Almagro et al. (2018)used manual assessments from 5816 subjects to validate their QC system,Esteban et al. (2017)used 1102 subjects in ABIDE dataset to train MRIQC model, andPizarro et al. (2016)used 1457 subjects from their dataset to build SVM QC classifier. Various other studies used deep learning approaches to classify the scans as pass or fail using the entire image instead of specific quality measures (Bottani et al., 2022;Keshavan et al., 2019). Tools for brain morphometric analysis such as Computational Anatomy Toolbox (CAT12) also offer quality control ratings based on tissue segmentation to evaluate scan quality (Gaser et al., 2024). Despite the availability of these methods using heterogeneous brain scans, many of them lack generalisability, as they focus majorly on research datasets from healthy subjects, specific acquisition protocol (Alfaro-Almagro et al., 2018), population (Esteban et al., 2017;Mortamet et al., 2009), or processing tools (Klapwijk et al., 2019), with very few exceptions on utilising routine clinical data from data warehouse (Bottani et al., 2022). To perform successful quality control in large clinical research datasets, it is important to establish a framework that offers broader applicability across various clinical cohorts, age range, and scanner types.

In this study, we tested two existing automated QC tools: MRIQC and CAT12. MRIQC is an open-source tool, offering an extensive array of metrics for evaluating quality on raw T1w images (based on noise, information theory, and specific artefacts), and it has become a standard reference in numerous studies (Chen et al., 2023;Elliott et al., 2023;Lorenzini et al., 2022). CAT12 is widely utilised in the field and encompasses a variety of quality control options (based on noise contrast, inhomogeneity contrast, resolution) applicable to images processed within the tissue segmentation pipeline (Besteher et al., 2022;Hahn et al., 2022;Sakreida et al., 2022). To classify the scans into pass or fail, MRIQC additionally provides a pre-trained supervised classifier which can be utilised to predict the quality of scans. In contrast, CAT12 provides image quality ratings for each measure which can be used to determine usable or unusable scans from the analysis. Due to their wide use and broad range of comprehensive measures available in both tools from raw and tissue-segmented scans, we selected these tools as good candidates to perform QC on clinical research datasets. We first tested the agreement between MRIQC and CAT12 with visual quality inspection on a large sample of clinical research data (*N*= 2438) from an extensive spectrum of datasets spanning ageing and neurodegenerative cohorts. We studied the relationship between the QC metrics produced by the two tools and tested the tools’ performance when adjusting the accept–reject threshold. We then proposed a new classification framework which uses a combination of QC metrics from both automated tools as features and visual QC as gold standard. We tested the generalisability of the proposed classifier on various test datasets that differed in terms of population and scanner. Finally, by looking at the distribution of QC measures that contributed most to the higher classification accuracy, we explored how they could be used to inform data harmonisation strategies. The outcomes of this study will be made accessible on the DPUK data portal, to support future clinical research studies.

## 2 Methods

### 2.1 Data and visual QC of T1w brain scans

The datasets used in this study are derived from established clinical research cohorts. These datasets were collected for research purposes, not as part of direct patient care or clinical trials. Therefore, we refer to them as clinical research datasets to reflect their use in research studies rather than routine clinical practice. Structural T1w brain images from 4 clinical research datasets (*N*= 2438) acquired on 39 scanners from 3 different manufacturers (Siemens, Philips, GE) were used: (1) Oxford Brain Health Clinic (BHC) (Griffanti et al., 2022) [age range: 65–101 years]; The BHC Research Database was reviewed and approved by the South Central—Oxford C research ethics committee (SC/19/0404). (2) Oxford Parkinson’s Disease Centre (OPDC) (Griffanti et al., 2020) [age range: 39–88 years]; The OPDC study was undertaken with the understanding and written consent of each subject, with the approval of the local NHS ethics committee (National Research Ethics Service (NRES) Committee South Central—Oxford A (Ref: 16/SC/0108) and Oxford C (Ref: 15/SC/0117); Berkshire Research Ethics Committee (Ref: 10/H0505/71)), and in compliance with national legislation and the Declaration of Helsinki. (3) Whitehall 2 imaging study (Filippini et al., 2014) [age range: 60–85 years]; ethical approval was granted generically for the “Protocol for non-invasive magnetic resonance investigations in healthy volunteers” (MSD/IDREC/2010/P17.2) by the University of Oxford Central University/Medical Science Division Interdisciplinary Research Ethics Committee (CUREC/MSD-IDREC), who also approved the specific protocol: “Predicting MRI abnormalities with longitudinal data of the Whitehall 2 sub-study” (MSD-IDREC-C1-2011-71). (4) Alzheimer’s Disease Neuroimaging Initiative (ADNI) (Petersen et al., 2010) [age range: 55–92 years]; As per ADNI protocols, all procedures performed in studies involving human participants were in accordance with the ethical standards of the institutional and/or national research committee and with the 1964 Helsinki Declaration and its later amendments or comparable ethical standards. The ADNI data collection was carried out after obtaining written informed consent from the participants. More details can be found atadni.loni.usc.edu.

Information on scanner, manufacturing model, counts, acquisition matrix, and voxel size for these datasets is provided inTable 1.

**Table 1.** Dataset-wise and scanner-wise counts of T1w scans of datasets used in this study.

Dataset	Sequence	Scanner	Field strength	Model	T1w Count	No. of slices	Voxel size
BHC	MPRAGE	Siemens	3T	Prisma	160	208	1 x 1 x 1
OPDC	MPRAGE	Siemens	3T	Trio	383	174	1 x 1 x 1
Whitehall1	Multi-Echo	Siemens	3T	Verio	552	176	1 x 1 x 1
	MPRAGE						
Whitehall2	Multi-Echo	Siemens	3T	Prisma	223	174	1 x 1 x 1
	MPRAGE						
ADNI	MPRAGE	Siemens	3T	Allegra	12	160	1 x 1 x 1.2
				Biograph_mMR	9	176	1 x 1 x 1, 1 x 1 x 1.2
				Prisma	27	208	
				Prisma_fit	82	175, 176, 208, 240	1 x 1 x 1, 1 x 1 x 1.2
				Skyra	41	176, 208	1 x 1 x 1, 1 x 1 x 1.2
				Skyra_fit	8	160, 176	1 x 1 x 1
				Trio	15	160	1 x 1 x 1.2
				TripTim	132	110, 160, 176	1 x 1 x 1, 1 x 1 x 1.2
				Verio	99	176	1 x 1 x 1, 1 x 1 x 1.2
			1.5T	Sonata	25	78, 160	1 x 1 x 1.2
				SonataVision	3	160	1 x 1 x 1.2
				Symphony	72	23, 145, 160	1 x 1 x 1.2, 1 x 1 x 3
				SymphonyTim	15	23, 160	1 x 1 x 1.2, 1 x 1 x 3
				Avanto	54	160, 176	1 x 1 x 1.2
				Espree	2	160	1 x 1 x 1.2
				NUMARIS/4	1	160	1 x 1 x 1.2
			2.9T	Allegra	7	160	1 x 1 x 1.2
				Trio	6	160	1 x 1 x 1.2
		GE	3T	GENESIS_SIGNA	3	166	1 x 1 x 1.2
				SIGNA_EXCITE	10	166	1 x 1 x 1.2
				SIGNA_HDx	11	166	1 x 1 x 1.2
			1.5T	GENESIS_SIGNA	34	180	1 x 1 x 1.2
				SIGNA_EXCITE	129	166, 180	1 x 1 x 1.2
				SIGNA_HDx	47	32, 166, 180	1 x 1 x 1.2
				Signa_HDxt	14	166	1 x 1 x 1.2
		Philips	3T	Achieva	93	170, 211	1 x 1 x 1, 1 x 1 x 1.2
				Achieva dStream	16	170, 211	1 x 1 x 1, 1 x 1 x 1.2
				GEMINI	6	170	1 x 1 x 1.2
				Ingenia	31	170, 211	1 x 1 x 1, 1 x 1 x 1.2
				Ingenuity	5	170	1 x 1 x 1.2
				Intera	49	170, 211	1 x 1 x 1, 1 x 1 x 1.2
			1.5T	Achieva	9	170	1 x 1 x 1.2
				Gyroscan Inera	1	170	1 x 1 x 1.2
				Gyroscan NT	3	170	1 x 1 x 1.2
				Intera	49	150, 170, 184	1 x 1 x 1.2

#### 2.1.1 Oxford Brain Health Clinic—BHC (N = 160)

The Oxford BHC is a joint clinical-research service for memory clinic patients which offers high-quality assessments not routinely available, including a multimodal brain MRI scan (Griffanti et al., 2022). Images are acquired on a Siemens 3T Prisma scanner using a protocol matched with the UK Biobank imaging study (Miller et al., 2016).

##### 2.1.1.1 Visual QC ratings

One of four raters visually inspected the raw images to identify low-quality scans to give an indication of tolerability of the protocol (e.g., motion artefacts that would be indicative of the ability of a memory clinic patient to lay still in the scanner), as well as to identify scans that might be informative about robustness in evaluating the analysis pipeline. The four raters followed similar guidelines reaching consensus in ambiguous cases.

The visual quality ratings were obtained from the dataset owners. These images were originally rated into low, medium, high quality. We categorised medium and high-quality images into accept label and low-quality images into reject label.

#### 2.1.2 Oxford Parkinson’s Disease Centre Discovery Cohort—OPDC (N = 383)

The OPDC study aims to identify biomarkers of Parkinson’s disease for early detection and progression. The dataset includes multimodal brain MRI data (acquired on a 3T Siemens Verio scanner) along with deep longitudinal clinical phenotyping in patients with Parkinson’s disease, at-risk individuals, and healthy elderly volunteers (Griffanti et al., 2020).

##### 2.1.2.1 Visual QC ratings

Scans were first checked ensuring that all the slices are acquired, and entire head is covered. Raw images were visually inspected to identify low-quality scans to give an indication of motion or scanner-related artefacts, as well as to identify scans that might be informative about robustness in evaluating the analysis pipeline.

For this dataset, the visual ratings were not available from dataset owners hence each image was visualised and rated into low, medium, and high quality by one rater. The medium- and high-quality images were grouped into accept category and low-quality images were grouped in reject category.

#### 2.1.3 Whitehall 2 imaging sub-study (N = 775)

The Whitehall 2 study is a longitudinal study of British civil servants to explore the factors affecting brain health and cognitive ageing (Filippini et al., 2014). In this dataset, 552 scans were acquired on a Siemens Verio 3T scanner (referred as Whitehall 1 in the manuscript—protocol details given inFilippini et al. (2014)and 223 scans on a Siemens Prisma 3T (referred as Whitehall 2 in the manuscript—protocol details given inZsoldos et al. (2020)). We treated the data from these two scanners separately in all the analyses for our work.

##### 2.1.3.1 Visual QC ratings

Images were manually inspected and classified as “low quality” if they contained evidence of excessive motion and/or scanner-related artefacts. Each scan was checked for motion and other artefacts by two independent analysts, and where there was disagreement, classification was decided through discussion.

The visual quality ratings (accept and reject) were obtained from the dataset owners.

#### 2.1.4 Alzheimer’s Disease Neuroimaging Initiative—ADNI (N = 1120)

The ADNI (adni.loni.usc.edu) was launched in 2003 as a public–private partnership, led by Principal Investigator Michael W. Weiner, MD. The primary goal of ADNI has been to test whether serial MRI, positron emission tomography (PET), other biological markers, and clinical and neuropsychological assessment can be combined to measure the progression of mild cognitive impairment (MCI) and early Alzheimer’s disease (AD). In this study, we included all the baseline T1w brain images from ADNI 1,2,3 and GO (first run in each session) (More details on the ADNI acquisition protocols is here:https://adni.loni.usc.edu/data-samples/adni-data/neuroimaging/mri/mri-scanner-protocols/). Due to the highly variable numbers of scans for each scanner, we grouped data from the same manufacturer and field strength together, for a total of seven ADNI sites.

##### 2.1.4.1 Visual QC ratings

Each series in each scan undergoes QC at the Mayo ADIR Lab. Trained analysts manually inspect images to ensure series-specific quality and assign a numerical grade to each scan: 1–3 is acceptable and 4 is failure (unusable).

- Check for adherence to the protocol parameters.
- Check for presence and severity of artefacts (e.g., participant motion)
- Check for anatomical coverage: ensuring that the entire head was imaged
- Check for completeness: all slices were acquired and transmitted
- Check for overall image quality

The visual quality ratings were available on a scale from*1*(excellent quality) to*4*(unusable). Upon careful inspection of the quality description, we decided to label images with a rating of 1 or 2 into the accept category and those with a rating of 3 or 4 into the reject category.

### 2.2 T1w processing in automated tools

All the images were named and organised in Brain Imaging Data Structure (BIDS) (Gorgolewski et al., 2016) and defaced (to preserve the privacy of individuals) before processing.

#### 2.2.1 MRIQC pipeline

MRIQC is an open-source pipeline that extracts image quality metrics (IQMs) from structural (T1w and T2w) and functional MRI data (Esteban et al., 2017). It uses modular sub-workflows from neuroimaging software toolboxes such as*FSL*(Jenkinson et al., 2012),*ANTs*(Avants BB et al., 2013), and*AFNI*at the background (Cox & Hyde, 1997). MRIQC also provides a random forest classifier (mriqc_clf) pre-trained on 1102 T1w scans (17 sites) from the Autism Brain Imaging Data Exchange (ABIDE) dataset. The classifier generates probability value for each scan (range*0*–*1*), and any scan with probability more than or equal to 0.5 (default threshold) is categorised to reject label.

Each defaced T1w brain image was processed in MRIQC pipeline (singularity version 0.15.1). The list of image quality metrics (IQMs) and their description are provided inTable 2(a detailed explanation can be found in the user manual ofMRIQC). From each image, 68 metrics were extracted. We used MRIQC’s random forest classifier (mriqc_clf) and labelled images into binary accept and reject labels.

**Table 2.** List of MRIQC image quality metrics.

QC category	QC measure	Explanation	References
Noise measurements	Coefficient of joint variation (CJV)	Higher values indicate heavy head motion and large image non-uniformity artefacts	( Ganzetti et al., 2016 )
	Contrast-to-noise ratio (CNR)	Higher values indicate better separation of grey matter (GM) and white matter (WM) tissue distribution	( Magnotta et al., 2006 )
	Signal-to-noise ratio (SNR)	Calculated for each tissue class	
	Dietrich’s SNR (SNRd)	SNR calculated with air background as reference	( Dietrich et al., 2007 )
	Mortamet’s quality index 2 (QI2)	Goodness-of-fit on the air mask once the artefactual intensities are removed; lower values are better	( Mortamet et al., 2009 )
Specific artefacts	Intensity non-uniformity (INU)	Summary statistics of INU field by N4ITK; values away from zero indicate higher inhomogeneity	( Tustison et al., 2010 )
	Mortamet’s quality index 1 (QI1)	Ratio of proportion of voxels with artefacts normalised by background voxels; lower values are better	( Mortamet et al., 2009 )
	White matter to maximum intensity ratio (wm2max)	Detecting the hyper-intensity of the carotid vessels and fat by calculating the median intensity within WM mask over 95% percentile of the full intensity distribution; good values are around [0.6, 0.8]	
Information theory	Entropy focused criterion (EFC)	Higher values indicate more ghosting and blurring induced by head motion	( Atkinson et al., 1997 )
	Foreground to background energy ratio (FBER)	Higher values indicate better signal within the head relative to outside the head	( Zarrar et al., 2015 )
Other	Full width at half maximum (FWHM)	FWHM of the spatial distribution of intensity values in voxel units; higher values indicate blurrier images	( Forman et al., 1995 )
	Volume fraction (icvs_*)	intracranial volume (ICV) fractions of GM, WM, and cerebrospinal fluid (CSF)	
	Residual partial volumes (rpve_*)	rpve of GM, WM, CSF	
	Overlap with tissue probability maps (overlap_*_*)	Overlap of tissue probability maps of ICBM nonlinear asymmetric 2009c template and maps estimated from image	
	Summary statistics (summary_*_*)	Summary measures of each tissue class with respect to voxels in the background	

#### 2.2.2 CAT12 pipeline

CAT12 (Computational Anatomy Toolbox) is an extension of SPM12 covering diverse morphometric methods to provide computational anatomy (Gaser et al., 2024). CAT12 provides a retrospective QC framework for empirical quantification of image quality parameters.

Each defaced T1w brain image was processed in CAT12 segmentation pipeline (standalone version r2042 running on v93 of MATLAB compiler runtime). The surface processing option was enabled during the segmentation. Post-segmentation, CAT12 generates a segmentation report for each image and provides image quality ratings (IQRs) based on noise, resolution, bias. It then aggregates these ratings into 16 weighted IQR [range A+ (excellent) to F (unacceptable/failed)]. The rating was defined for a percentage range and a numerical scaling that allows mapping to nominal letters (described in detail in the CAT12 documentation:https://neuro-jena.github.io/cat12-help/#qc). In this study, to label the images into accept and reject class, each image with weighted IQR of C minus and below (selected as “default threshold”) was labelled into reject class. To label the images into accept and reject quality, each image with weighted IQR of C minus and below (selected as “default threshold”) was labelled into reject class.

For the proposed classifier design (in the later section), we considered additional quality measures which are not provided in the CAT12 visualisation report (i.e., metrics other than noise. resolution, bias, and weighted IQR) but are saved in the output of CAT12 segmentation (provided as,*cat_<subjdirname>.mat*). The description of all these quality measures is provided inTable 3, (a detailed explanation can be found in the user manual of CAT12:https://neuro-jena.github.io/cat12-help/#qc). From each image, 36 quality measures were extracted (Parekh, 2021).

**Table 3.** List of CAT12 image quality measures.

QC category	QC measure	Explanation	References
QC measures in CAT12 report	Noise contrast ratio (NCR)	Local standard deviation in the optimised WM segment and scaled by the minimum tissue contrast; graded from A+ (excellent quality to F unacceptable/failed quality)	( Dahnke et al., n.d .) ( Collins et al., 1998 ) ( Reuter et al., 2015 ; Winterburn et al., 2013 )
	Inhomogeneity contrast ratio (ICR)	Global standard deviation within the optimised WM segment and is scaled by the minimum tissue contrast; graded from A+ (excellent quality to F unacceptable/failed quality)	
	Root-mean-square resolution (RES)	Root-mean-square value of the voxel size; graded from A+ (excellent quality to F unacceptable/failed quality)	
	Weighted average image quality rating (IQR)	Average rating obtained from NCR, ICR, RES; graded from A+ (excellent quality to F unacceptable/failed quality)	
Other additional measures calculated after segmentation (added to classifier)			
Surface measures	Mean surface Euler number Mean surface defect number Mean surface defect area Surface intensity RMSE Surface position RMSE		( Dahnke et al., 2013 ; Yotter, Dahnke, et al., 2011 ; Yotter, Thompson, et al., 2011 )
Tissue measures	Absolute and relative mean & standard deviation of GM, WM, CSF tissue intensities Absolute and relative contrast between the tissue classes Absolute and relative volume of GM, WM, CSF tissues and WM hyperintensities		

### 2.3 Comparison of MRIQC and CAT12 quality measures

We first compared the quality measures between the two automated tools. The quality measures derived from MRIQC and CAT12 were correlated using Pearson’s correlation. The correlation analysis was conducted in MATLAB2022b (*The MathWorks Inc. (2022). MATLAB Version: 9.13.0.2105380 (R2022b)*, 2022).

### 2.4 Comparison of ratings between automated tools and visual QC

We calculated the percentage of scans that would pass QC and compared the agreement between visual QC, MRIQC classifier predictions (default threshold,*MRIQC(D)*), and CAT12’s weighted IQR (default threshold,*CAT12(D)*) using Kappa coefficient of inter-rater reliability (IRR) (Landis & Koch, 1977;McHugh, 2012). Three comparisons were performed: (1) CAT12 ratings versus MRIQC ratings, (2) CAT12 ratings versus visual QC ratings, (3) MRIQC ratings versus visual QC ratings.

Further, we explored the effect of changing the labelling threshold from MRIQC classifier and CAT12’s weighted IQR. We investigated this by changing the CAT12’s weighted IQR threshold to – (1) strict (*CAT12 (-)*): any scan with weighted IQR rating C and below were labelled to reject category, (2) lenient (*CAT12 (+)*): any scan with weighted IQR rating D+ and below were labelled to reject category. Similarly, for the MRIQC classifier, we changed the threshold of acceptance to – (1) strict (*MRIQC (-)*): scans with probability equal to or more than 0.4 were labelled to reject category, (2) lenient (*MRIQC (+)*): scans with probability equal to or more than 0.6 were labelled to reject category. We then re-calculated the Kappa coefficient for the above three comparisons. The Kappa coefficient was calculated using IRR package in R (Gamer et al., 2019;R Core Team, 2022).

### 2.5 Proposed QC classifier

In this section we present our proposed QC classifier. The primary model (combined data model) was trained and tested on a mix of data from multiple datasets and sites. We then tested the generalisability of our classification framework in a leave-one-site-out approach and in cases where training and test data differ in terms of field strength, scanner manufacturer, and study population.

#### 2.5.1 Combined data model

##### 2.5.1.1 Data and classifiers

We designed a binary QC classifier which combines the MRIQC and CAT12 quality measures as features. Binary visual QC ratings were used as target. For the combined data model, we first randomly divided our entire sample (*N*= 2438) into 80% training (*N*= 1955) and 20% test data (*N*= 483). The data were divided ensuring fair representation of target labels, sites, and proportion of patients and controls (when applicable) among both the training and test datasets. The site-wise and label-wise split for training and test datasets is provided inTable 4(Also, see full table with dataset, diagnosis, scanner, and label-wise counts inSupplementary Materials,Table S1). We tested three options for the underlying machine learning classification: support vector machine, random forest, and random under-sampling boost.

**Table 4.** Site-wise split of training and test data for the combined data model.

	Train data			Test data		
Datasets	Reject	Accept	Total	Reject	Accept	Total
ADNI GE 1.5T	8	172	180	1	43	44
ADNI GE 3T	4	16	20	0	4	4
ADNI Philips 1.5T	1	49	50	0	12	12
ADNI Philips 3T	7	153	160	2	38	40
ADNI Siemens 3T	8	332	340	2	83	85
ADNI Siemens 1.5T	10	128	138	2	32	34
ADNI Siemens 2.9T	2	9	11	0	2	2
OPDC Siemens 3T	47	260	307	12	64	76
BHC Siemens 3T	12	116	128	4	28	32
Whitehall 1 Siemens 3T	35	407	442	9	101	110
Whitehall 2 Siemens 3T	6	173	179	1	43	44
Total	140	1815	1955	33	450	483

Support vector machine (SVM) is one of the most common supervised classifiers, simple to train for hyperparameters, effectively handles high-dimensional data, and less prone to overfitting than non-linear classifiers (Cortes & Vapnik, 1995). We used the “fitcsvm” implementation in MATLAB (*MATLAB Version 9.14.0.2239454 (R2023a)*, 2023). Two hyperparameters were optimised in nested cross-validation (CV): box constraint (0.01, 0.1, 1, 10, 100, 1000) and Kernel function (linear, radial basis function). The remaining hyperparameters were maintained at their default settings. Random forest (RF) is a supervised classifier robust to outliers and non-linear data, faster to train, and handles unbalanced classes in the data (as in our data “reject” class, samples are substantially lower than “accept” class) (Breiman, 2001). We used the “fitcensemble” implementation in MATLAB (*MATLAB Version 9.14.0.2239454 (R2023a)*, 2023). Two hyperparameters were optimised in nested CV: maximal number of decision splits (10, 50) and number of ensemble learning cycles (10, 50, 100). The remaining hyperparameters were maintained at their default settings. We selected random under-sampling boost (RUS) as third classifier due to its ease of implementation, effective handling of imbalanced classes, rapid processing speed, and reduced computational complexity (Seiffert et al., 2008). It is a supervised classifier that under samples the majority class labels in the training process to balance the minority class. Given the imbalance of classes in our data, we used random under-sampling to avoid skewing towards the majority class (accept) and improve the detection of the minority class (reject) in our datasets. We used the “fitcensemble” implementation in MATLAB (*MATLAB Version 9.14.0.2239454 (R2023a)*, 2023). Three hyperparameters were optimised in nested CV: maximal number of decision splits (10, 50), number of ensemble learning cycles (10, 50, 100), and learning rate for shrinkage (0.01, 0.1). The remaining hyperparameters were maintained at their default settings.

##### 2.5.1.2 Nested cross-validation approach

The classifiers were trained in a nested cross-validation (CV) framework consisting five outer folds and three inner folds (seeFig 1). In the training phase, within every CV iteration, the first step is feature pre-processing where we performed site-wise z-score normalisation of features. During this procedure, only the mean and standard deviation from train data were applied to the test data to avoid the data leakage. For ranking of the features in the inner and outer folds, we chose univariate feature selection methods for their simplicity and ease of implementation as these methods allow for straightforward evaluation of each feature’s individual relevance to the target variable and are computationally efficient. However, to address the potential bias introduced by highly correlated features, we also employed multivariate methods such as ReliefF and Minimum Redundancy Maximum Relevance (MRMR) which can help mitigate redundancy by considering feature interactions and reducing the impact of correlations between features. The feature ranks from the multiple methods were then aggregated using robust ranking aggregation (Kolde et al., 2012). For each feature size (iterative; 10, 20, 30, 40, 50, 60, 70, 80, 90, 100, 104 (all)), the classifier was trained on the inner fold’s train data and tested on the inner fold’s test data for the grid of hyperparameters. For each outer CV iteration and for each feature size, the classification performance was averaged over all the inner CV folds and the combination of hyperparameters achieving the best performance was chosen. Finally, for each feature size, the outer cross-validation iteration was executed with the chosen combination of hyperparameters from the inner folds, models were re-trained, and tested on the outer test data. To get precise estimates of model’s performance, we ran a total 100 iterations of the nested CV in the training phase and obtained the best combination of hyperparameters for each feature size for each classifier. In the final model design, we aggregated feature ranks from all outer cross-validation folds across 100 iterations and derived a final ranking of the features. The final model was then trained by using all the training data with the best combination of hyperparameters for each feature size and feature ranking across 100 CV iterations. For the final model, z-score normalisation was applied on entire training data and only the mean and standard deviation from training data were applied to the holdout test data.

**Fig. 1.**
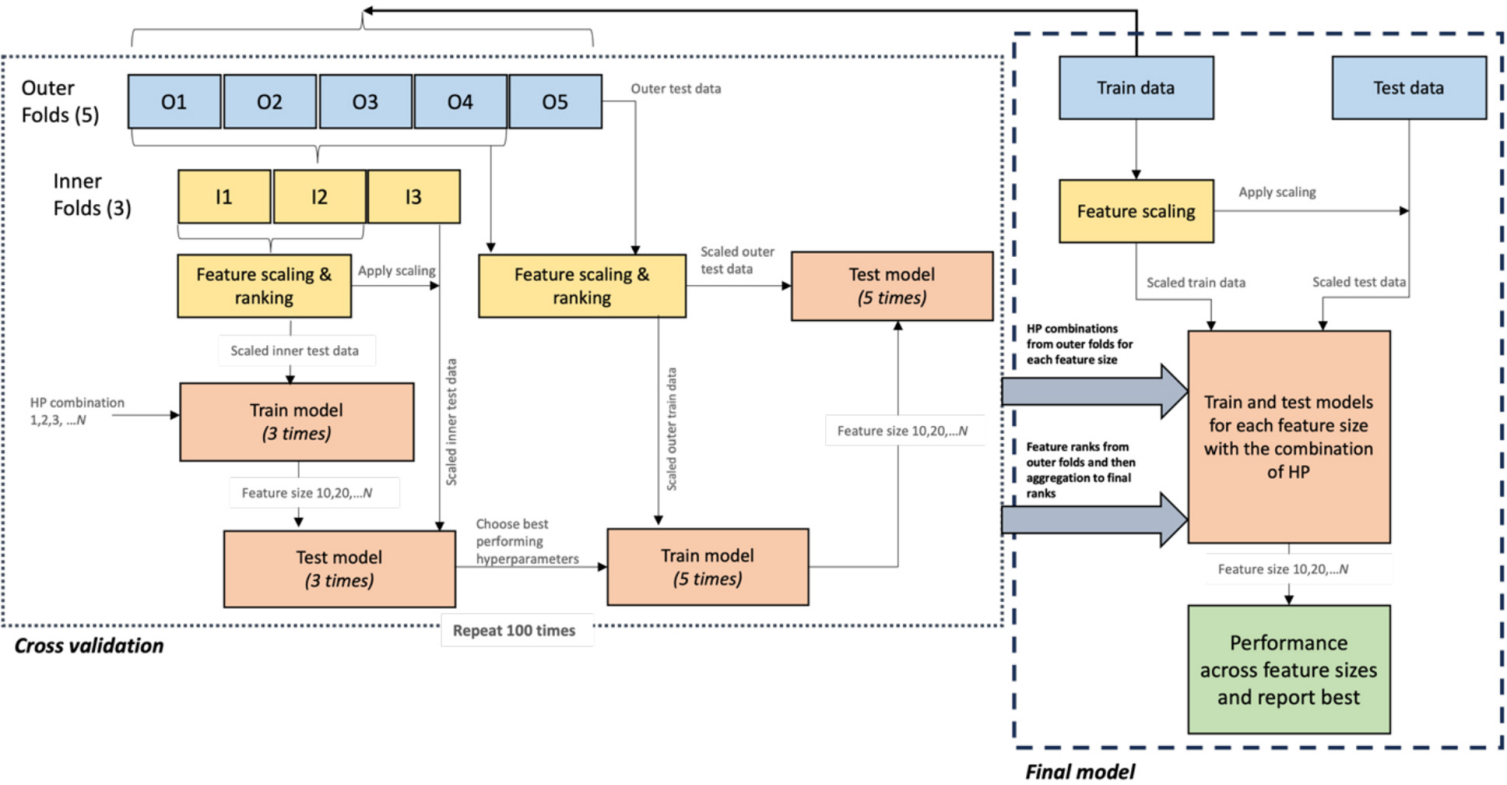
Nested cross-validation workflow for training the QC classifier. The model was trained for five outer folds and three inner folds. The hyperparameters of the model were optimised on the inner test data, and the combination yielding the highest balanced accuracy was selected for evaluation on the outer folds. The nested cross-validation process was repeated 100 times. The best performing hyperparameters for each feature size and feature ranks across 100 iterations were used to train the final model and tested on the hold-out data.

##### 2.5.1.3 Assessment and comparison of prediction performance

The performance of the models was evaluated using various metrics, including true positive rate, false positive rate, and balanced accuracy (Eqs. 1–4).

Sensitivity(True positive rate)=TP​/​(TP+FN). (1)

Specificity=TN ​/​(TN+FP) (2)

Balanced accuracy=(Sensitivity+Specificity)​/​2 (3)

False positive rate=FP​/​(FP+TN) (4)

True positive (TP)—the model correctly predicts the accept label; true negative (TN)—correctly predicts reject label; false positive (FP)—wrongly predicts accept label for a scan that should be rejected; false negative (FN)—wrongly predicts reject label for a scan that should be accepted.

For the purpose of model selection and comparison of classifier performance on the test data, balanced accuracy was employed as the primary evaluation metric. The choice to use balanced accuracy as our primary metric is based on the fact that our datasets have imbalance in the accept and reject classes and we are interested in both classes being predicted well for unseen datasets.

For each classifier (SVM, RF, RUS), we selected the feature size that gave the best performance. We then compared the prediction performance of the three optimised classifiers with each other and with MRIQC and CAT12. This comparison of prediction performance was done for (1) combined test data (*N*= 483), (2) test data categorised by site (seeTable 4for number of scans in each site in each class), (3) patients and controls separately within the test data (seeTable 5for number of scans for in each class), (4) each sub-category of diagnosis within the test data (seeTable 5for number of scans in each class for each category).

**Table 5.** Diagnosis group-wise number of scans in accept and reject labels.

	Diagnosis group	Train Reject	Train Accept	Train Total	Test Reject	Test Accept	Test Total
Controls	ADNI	13	252	265	2	71	73
	OPDC	8	66	74	2	14	16
	Whitehall 1	35	407	442	9	101	110
	Whitehall 2	6	173	179	1	43	44
	Total	62	898	960	14	229	243
Patients	ADNI Dementia	16	148	164	2	26	28
	ADNI MCI	11	459	470	3	117	120
	OPDC RBD	16	81	97	4	23	27
	OPDC iPD	22	113	135	6	27	33
	BHC	12	116	128	4	28	32
	Total	77	917	994	19	221	240

MCI: mild cognitive impairment, RBD: REM sleep behaviour disorder (at-risk group for PD), iPD: idiopathic Parkinson’s disease.

##### 2.5.1.4 Feature importance

We investigated the distribution of the top 10 ranked features derived from the final combined data model by employing kernel density and scatter plots. To ascertain potential statistical variations in the distribution among sites, we conducted a two-sample Kolmogorov–Smirnov test. Subsequently, to address multiple comparisons, we applied the Bonferroni correction to obtain adjusted p-values.

#### 2.5.2 Additional validation of combined data model

To ensure our combined data model is robust and effective across heterogeneous datasets, sites, and scanners, it is crucial to evaluate how well it generalises to different scanners and sites. Hence, we created leave-one-site-out and exploratory models as explained in the sections below. From combined data model, we extracted the combinations of hyperparameters, feature ranking and feature size at which the best performance was observed on the combined test data. These parameters were then used to re-train classifier on different training datasets (see belowTable 6for leave-one-site-out models,Tables 7and8for exploratory models). During this process, z-score normalisation was applied on entire training data (which does not include any data from the test site) and only the mean and standard deviation from training data were applied to the test site. This approach does not consider re-training the models from scratch but allows us to test the combined data model on different test datasets. However, for comparison, we also repeated leave-one-site-out and exploratory model training from scratch (to avoid data leakage) and provided the results in theSupplementary Materials(Supplementary Analysis 2).

**Table 6.** Train and test split for leave-one-site-out models.

Test Dataset	Train Reject	Train Accept	Train Total	Test Reject	Test Accept	Test Total
ADNI GE 1.5T	132	1643	1775	9	215	224
ADNI GE 3T	136	1799	1935	4	20	24
ADNI Philips 1.5T	139	1766	1905	1	61	62
ADNI Philips 3T	133	1662	1795	9	191	200
ADNI Siemens 3T	132	1483	1615	10	415	425
ADNI Siemens 1.5T	130	1687	1817	12	160	172
ADNI Siemens 2.9T	138	1806	1944	2	11	13
OPDC Siemens 3T	93	1555	1648	59	324	383
BHC Siemens 3T	128	1699	1827	16	144	160
Whitehall 1 Siemens 3T	105	1408	1513	44	508	552
Whitehall 2 Siemens 3T	134	1642	1776	7	216	223

**Table 7.** Training and test data split for exploratory models on field strengths and manufacturers.

Models	Training sites	*N* training—Total (accept)	Test dataset	*N* test—Total (accept)
Generalisability across field strength	3T (Siemens, Philips, GE)	1576 (1457)	Siemens 1.5T, Philips 1.5T, GE 1.5T	458 (436)
Generalisability across manufacturer	3T, 2.9T, 1.5T (Siemens)	1545 (1425)	3T (Philips, GE), 1.5T (Philips, GE)	510 (487)
Generalisability across manufacturer and field strength	3T (Siemens)	1396 (1288)	3T (Philips, GE), 1.5T (Siemens, Philips, GE), 2.9T (Siemens)	695 (658)

##### 2.5.2.1 Leave-one-site-out models

We created leave-one-site-out CV models using the best performing classifier on the combined data (among SVM, RF, and RUS) to see how well our training workflow generalises to an unseen site. From this classifier, we extracted the combinations of hyperparameters, feature ranking and feature size at which the best performance was observed on the combined test data. These parameters were then used to re-train classifier on data from remaining sites while keeping each site as test data. Finally, the classification performance on each test site was assessed, comparing them against MRIQC and CAT12, and against the best performance of a combined data model on each site in the test data. The split of data for training and testing for each model is provided inTable 6.

##### 2.5.2.2 Exploratory models

Finally, we explored how well our approach generalises when the model is trained on data from one field strength and/or manufacturer and tested on data from other field strengths/manufacturers. These exploratory models were designed to test:

1. Generalisability across field strength: the majority of the datasets were acquired on 3T scanners (*N*= 1967), hence we trained the model on data from all 3T scanners and tested on the data from 1.5T field strengths.
2. Generalisability across manufacturer: the majority of the datasets were acquired from Siemens scanners (*N*= 1928), hence we trained the model combining Siemens data from all field strengths and tested on data from other manufacturers.
3. Generalisability across manufacturer and field strength: the majority of the data are from 3T Siemens scanners (*N*= 1743), hence we trained the model only from 3T Siemens scanner data and tested on the remaining data.
4. Generalisability to a movement-related artefacts dataset: To test whether the proposed classifier could be used also on a very different population to those originally trained with, in terms of both demographics and level of artefacts, we used the Movement-Related ARTefacts (MR-ART) dataset (Nárai et al., 2022). Briefly, this dataset was designed to include both motion-free (N = 148) and motion-affected data (N = 288) acquired from the same young healthy participants (age mean age 30 ± 13 years). We tested the combined data model directly on the MR-ART dataset, as well as the option of incorporating samples from MR-ART in the training data (between 1% and 90%). For more details on this analysis, refer toSupplementary Analysis 3.

The data split for training and test data on the first three models is provided inTable 7, whileTable 8illustrates the equivalent details for the models using the MR-ART dataset. Similar to the leave-one-site-out models, we chose the best performing classifier (among SVM, RF, and RUS) on the combined test data and re-trained and tested the classifier for the different cases. As explained above, the training process used hyperparameter combinations, feature ranking and feature size at which the best performance was observed on the combined test data. The classification performances were assessed for each model, comparing them against MRIQC and CAT12, and against the performance of the combined data model.

**Table 8.** Training and testing split for exploratory models on the MR-ART dataset.

Models	Training datasets	N training—Total (accept)	MR-ART N training—Total (accept)	MR-ART N test—Total (accept)
Combined data model	All main datasets ( Table 4 )	1955 (1815)	0	436 (238)
1% MR-ART	All main datasets ( Table 4 ) and 1% MR-ART samples	1961 (1819)	6 (4)	430 (234)
5% MR-ART	All main datasets ( Table 4 ) and 5% MR-ART samples	1979 (1829)	24 (14)	412 (224)
30% MR-ART	All main datasets ( Table 4 ) and 30% MR-ART samples	2088 (1888)	133 (73)	303 (165)
60% MR-ART	All main datasets ( Table 4 ) and 60% MR-ART samples	2131 (1911)	263 (144)	173 (94)
80% MR-ART	All main datasets ( Table 4 ) and 80% MR-ART samples	2305 (2006)	350 (191)	86 (47)
90% MR-ART	All main datasets ( Table 4 ) and 90% MR-ART samples	2349 (2031)	394 (216)	42 (22)

## 3 Results

### 3.1 Comparison of quality measures (CAT12 vs. MRIQC)

**Fig. 2.**
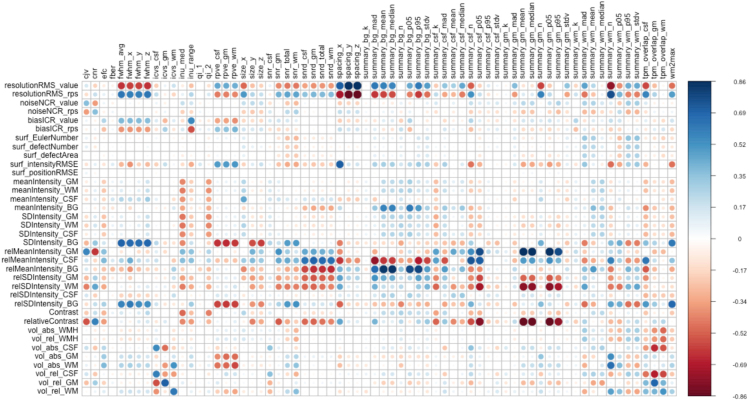
Correlation plot between MRIQC IQMS (columns) and CAT12 quality measures (rows). MRIQC generated total 68 IQMs and from CAT12 we extracted 36 quality measures.

We analysed the correlation between CAT12 quality measures and MRIQC IQMs (Fig. 2) to explore both common and distinct metrics within these tools. We observed various statistically significant correlation coefficients between pairs of measures from these automated tools. For instance, CAT12’s resolution measure exhibited significant correlation with MRIQC’s summary-based metrics derived from tissues, FWHM, image size, image spacing, and overlap of CSF with tissue probability maps (TPM). The absolute volume of tissues measured by CAT12 demonstrated significant correlations with MRIQC’s intra-cranial volume fraction of tissues and overlap of tissue classes with TPM. The relative intensity of background in CAT12 exhibited a significant correlation with MRIQC measures encompassing noise-based metrics, measures tied to specific artefacts (such as image nonuniformity), as well as other parameters such as size, spacing, FWHM, and residual partial volumes of tissues. Relative intensities from CSF and GM in CAT12 were significantly correlated with summary measures from background, CSF, and GM in MRIQC. Summary measures derived from WM in MRIQC significantly correlated with CAT12’s resolution, relative intensity of CSF, and absolute volumes of GM and WM tissues. Similarly, CAT12’s relative contrast showed a significant correlation with summary measures from CSF and GM in MRIQC. However, non-significant or low correlations (below ±0.5) suggest that the two tools are also capturing unique information about the image. Measures falling in this category for CAT12 are noise, mean intensity from tissues, and surface measures while for MRIQC are QI1, QI2 (targeting specific artefacts), EFC, FBER (informed by information theory). For detailed correlation coefficients, p-values, and the upper and lower bounds of a 95% confidence interval for each pair of measures, refer toSupplementary Excel file(sheet name: “correlations”).

### 3.2 Comparison of ratings between automated tools and visual QC

#### 3.2.1 Percentage of scans passing QC

We first compared the percentage of scans that passed (accept category) QC using visual QC, MRIQC, and CAT12. The results are reported inTable 9. Overall, CAT12 showed the highest acceptance percentage and MRIQC classifier showed the lowest acceptance percentage.

**Table 9.** Percentage of scans passing visual QC and QC from automated tools.

Dataset	Field Strength	Scanner	Total N scans available	% accept visual QC	% accept MRIQC (MRIQC—Visual)	% accept CAT12 QC (CAT12—visual)
ADNI	1.5T	GE	224	96	96 (0)	98.7 (2.7)
ADNI	3T	GE	24	83.3	91.7 (8.4)	95.8 (12.5)
ADNI	1.5T	Philips	62	98.4	90.3 (-8.1)	100 (1.6)
ADNI	3T	Philips	200	95.5	88 (-7.5)	98 (2.5)
ADNI	3T	Siemens	425	97.6	94.1 (-3.5)	98.8 (1.2)
ADNI	1.5T	Siemens	172	93	89 (-7.7)	98.8 (5.8)
ADNI	2.9T	Siemens	13	84.6	76.9 (-7.7)	100 (15.4)
OPDC	3T	Siemens	383	84.6	91.4 (6.8)	98.7 (14.1)
BHC	3T	Siemens	160	90	80 (-10)	91.9 (1.9)
Whitehall 1	3T	Siemens	552	92	92.9 (0.9)	94.6 (2.6)
Whitehall 2	3T	Siemens	223	96.9	96.4 (-0.5)	98.7 (1.8)
All datasets			2438	92.9	91.8 (-1.1)	97.3 (4.4)

#### 3.2.2 Classification agreement

**Fig. 3.**
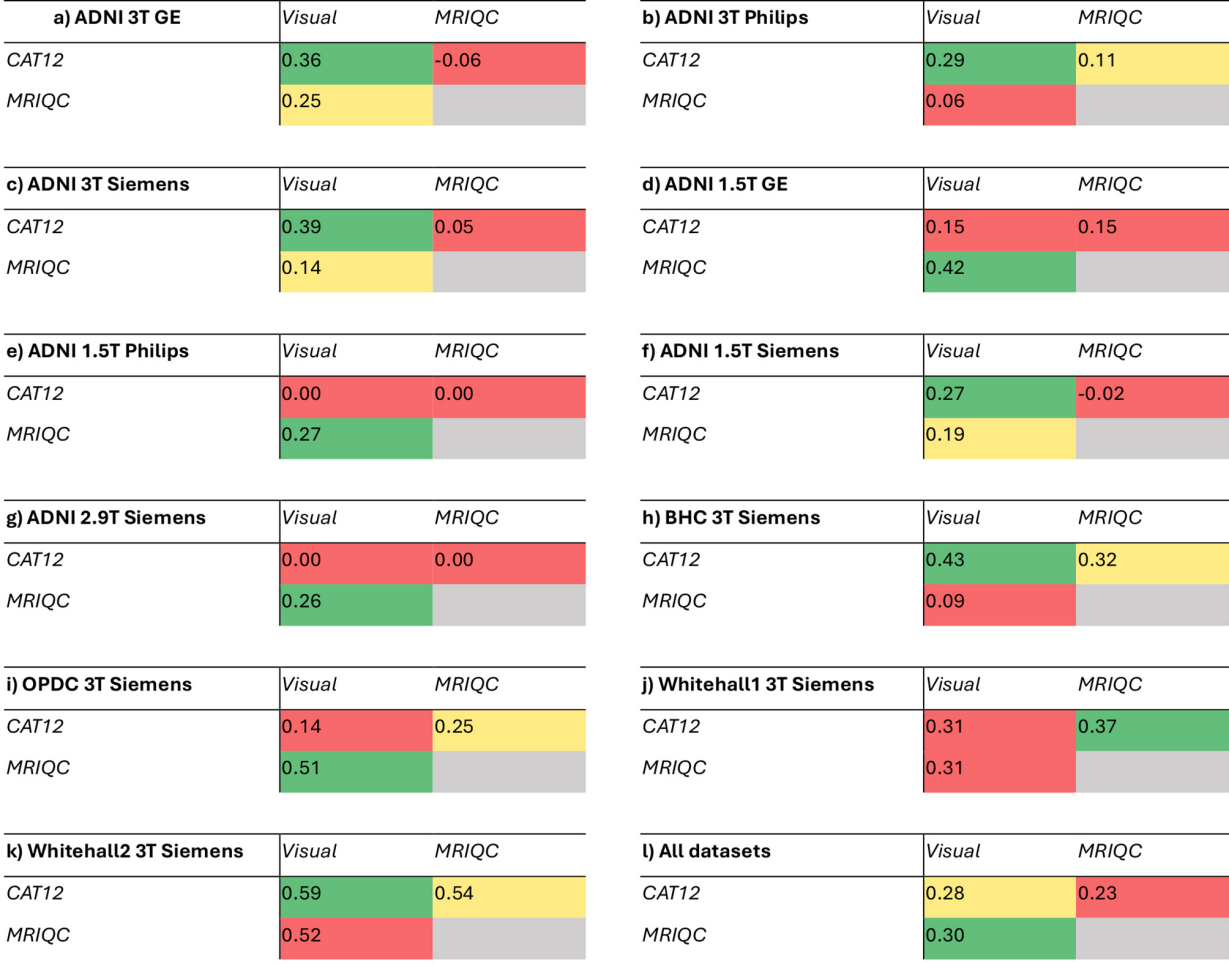
Kappa coefficient values comparing the agreement of ratings between visual QC and automated tools (panels a-k illustrate results for each dataset separately, panel l for all datasets combined). The colours indicate the lowest (red), medium (yellow), and highest values (green) in each dataset. SeeSupplementary Excel filefor details (sheet name—“IRR”).

We computed Kappa coefficient to measure the agreement between the automated tools and with visual QC (Fig. 3). A detailed table of Kappa coefficients, associated p-values, and percentage agreement for all the pairs of ratings is provided inSupplementary Excel file(sheet name—“IRR”).

##### 3.2.2.1 Visual QC versus automated tools

When evaluating the agreement on all datasets together (Fig. 3, panel l), MRIQC and visual QC showed higher value of Kappa coefficient (k = 0.3) than CAT12 and visual QC (k = 0.28). However, when looking at each dataset separately, in some cases the agreement was higher between CAT12 and visual QC (panels a, b, c, f, h, k, Kappa between 0.27 and 0.59), while other datasets showed higher Kappa coefficient between MRIQC and visual QC (panels d, e, g, i, j, Kappa between 0.26 and 0.51). Notably, ADNI 1.5T Philips and 2.9T Siemens showed no agreement between CAT12 and visual QC (panels e, g).

##### 3.2.2.2 CAT12 versus MRIQC ratings

For all datasets together, we found significant agreement between the ratings from CAT12 and MRIQC ratings (k = 0.23). When considered each dataset separately, Whitehall 2 dataset showed the highest agreement (k = 0.54). Notably, some datasets in ADNI (3T GE, 1.5T Siemens, 1.5T Philips, 2.9T Siemens) showed no agreement or worse than expected agreement (zero or negative values of Kappa coefficient inFig. 3, panels a, f, e, g).

#### 3.2.3 Impact of threshold on classification agreement

Given that the inter-rater reliability did not show consistency across datasets on which tool produced more similar ratings to visual QC using their default threshold (0.28 for CAT12, 0.30 for MRIQC), we explored the effect of using a lenient and stricter threshold of acceptance on the automated tools. The percentage of accepted scans upon adjusting the threshold is provided inTable 10. A detailed table of Kappa coefficients, associated p-values, and percentage agreement for all the pairs of ratings is provided inSupplementary Excel file(sheet name—“kappaCoefficient_agreement”).

**Table 10.** Percentage of accepted scans after adjusting acceptance thresholds for MRIQC and CAT12 (“-” for strict threshold and “+” for lenient threshold).

Dataset	Field Strength	Scanner	Total scans	% accept visual QC	% accept CAT12 (-) (CAT12 (−)—Visual QC)	% accept CAT12 (+) (CAT12 (+)—Visual QC)	% accept MRIQC (-) (MRIQC (−)—Visual QC)	% accept MRIQC (+) (MRIQC (+)—Visual QC)
ADNI	1.5T	GE	224	96	95.1 (1.1)	100 (4)	86.6 (-10.6)	97.8 (1.8)
ADNI	3T	GE	24	83.3	91.7 (8.4)	100 (16.7)	87.5 (4.2)	95.8 (12.5)
ADNI	1.5T	Philips	62	98.4	95.2 (-3.2)	100 (1.6)	85.5 (-12.9)	93.5 (-4.9)
ADNI	3T	Philips	200	95.5	94 (-1.5)	99 (3.5)	78 (-17.5)	93.5 (-2)
ADNI	3T	Siemens	425	97.6	96.9 (-0.7)	99.5 (1.9)	83.5 (-14.1)	97.2 (-0.4)
ADNI	1.5T	Siemens	172	93	96.5 (3.5)	100 (7)	77.3 (-15.7)	97.7 (4.7)
ADNI	2.9T	Siemens	13	84.6	100 (15.4)	100 (15.4)	76.9 (-7.7)	84.6 (0)
OPDC	3T	Siemens	383	84.6	95.6 (11)	99.2 (14.6)	69.7 (-14.9)	94.5 (9.9)
BHC	3T	Siemens	160	90	79.4 (-10.6)	96.9 (6.9)	65 (-25)	91.3 (1.3)
Whitehall1	3T	Siemens	552	92	88.2 (-3.8)	98.2 (6.2)	75.7 (-16.3)	97.3 (5.3)
Whitehall2	3T	Siemens	223	96.9	96 (-0.9)	99.6 (2.7)	61.4 (-35.5)	98.2 (1.3)
All datasets			2438	92.9	93 (0.1)	99 (6)	75.8 (-17.1)	96.1 (3.2)

##### 3.2.3.1 Visual QC versus automated tools

We recalculated the Kappa coefficient values after adjustment of thresholds to find the agreement between the automated tools and visual QC ratings (seeFig. 4). When looking at the Kappa coefficient values from all datasets together (panel l), we found that the agreement between visual QC and CAT12 ratings improved after applying a strict threshold to CAT12. We found a similar effect when looking at each dataset separately on most of our datasets (panels b, d, e, h, I, j). For example, the lack of agreement between visual QC ratings and default threshold ratings of CAT12 (k = 0) for ADNI 1.5T Philips dataset improved significantly (k = 0.43) after applying strict threshold to CAT12 ratings. However, some datasets did not show any improvement in Kappa coefficient after adjusting thresholds (panels a, c, g, k).

**Fig. 4.**
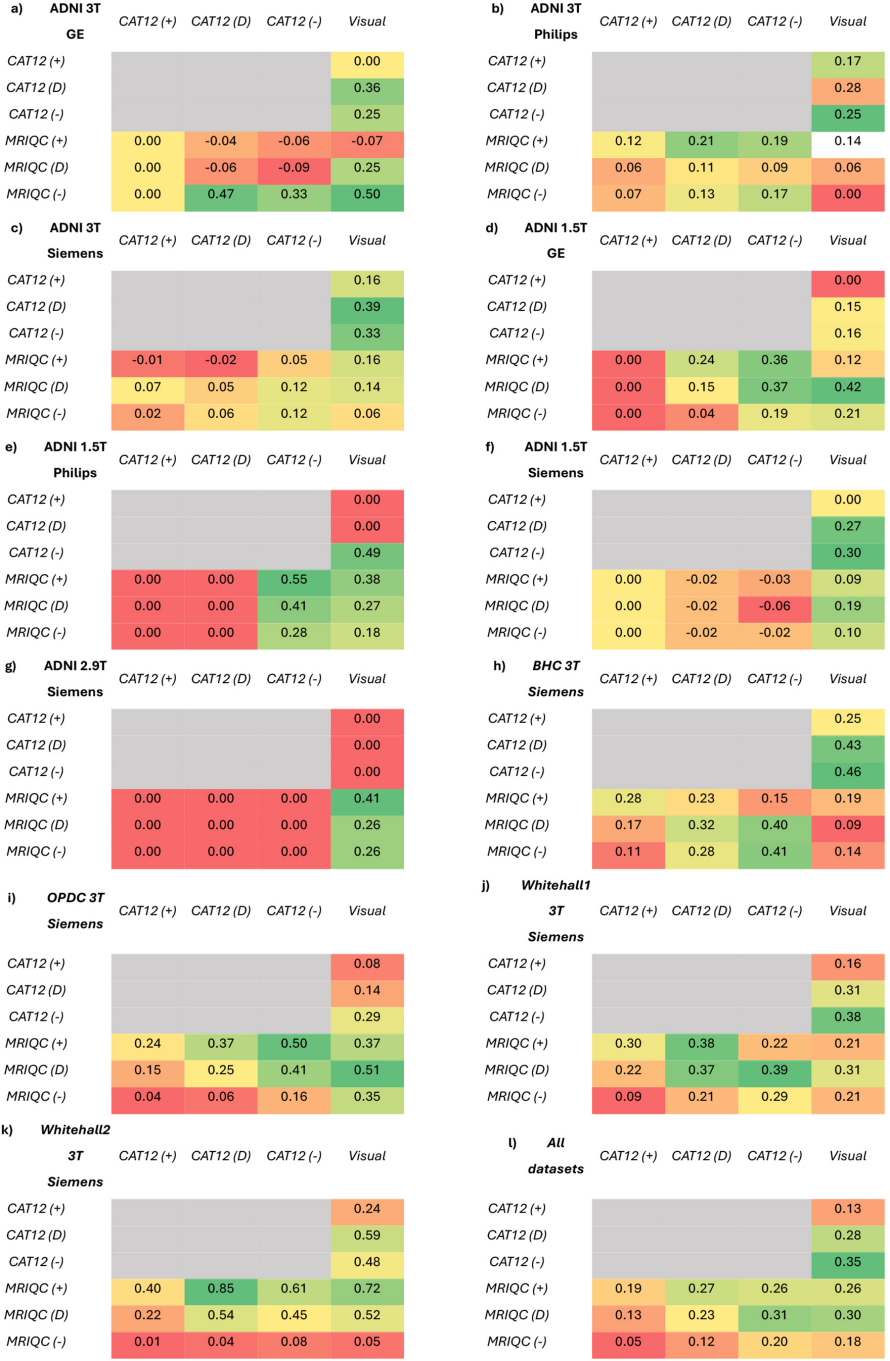
Kappa coefficient values comparing the agreement of ratings between visual QC and automated tools after adjusting the acceptance thresholds of automated tools (panels a-k illustrate results for each dataset separately, panel l for all datasets combined). The comparison with default thresholds (D) is also provided for ease of comparison. The colours indicate the lowest (red), medium (yellow), and highest values (green) in each panel. SeeSupplementary Excel filefor details (sheet—“IRR”).

For all datasets together, we did not see any improvement in Kappa coefficient when comparing visual QC with changed thresholds in MRIQC ratings. Some datasets showed increased agreement after applying lenient threshold to MRIQC ratings (panels b, c, e, g, h, k). For example, the significant agreement between visual QC and default threshold ratings of MRIQC in Whitehall 2 (k = 0.52) was further improved (k = 0.72) after applying lenient threshold to MRIQC ratings. Only ADNI 1.5T GE dataset showed significantly improved value of Kappa coefficient after applying strict threshold to MRIQC ratings (from k = 0.25 to k = 0.5). The rest of the datasets did not show any improvement upon adjusting the threshold of MRIQC ratings (panels d, f, i, j).

##### 3.2.3.2 CAT12 versus MRIQC ratings

For all datasets together and each dataset separately, the Kappa coefficient significantly improved between MRIQC default threshold ratings and CAT12 ratings after applying a strict threshold (from k = 0.23 to k = 0.31). Most of the datasets showed similar effect of improvement between default threshold ratings of MRIQC and CAT12 ratings after applying strict threshold (panels c, d, e, h, i, j). For Whitehall2 and ADNI 3T Phillips, the Kappa coefficient improved between default ratings of CAT12 and MRIQC ratings after applying lenient threshold (for Whitehall 2 - from 0.54 to 0.85; for ADNI 3T Phillips - from 0.11 to 0.21). Only for ADNI 3T GE dataset, the Kappa coefficient between default threshold ratings of CAT12 and MRIQC improved (from k = -0.06 to 0.47) after applying strict threshold to MRIQC ratings. Notably, ADNI 1.5T Siemens and ADNI 2.9T Siemens did not show any improvement upon adjustment of thresholds, showing zero agreement.

### 3.3 Classification performance

#### 3.3.1 Combined data model

**Fig. 5.**
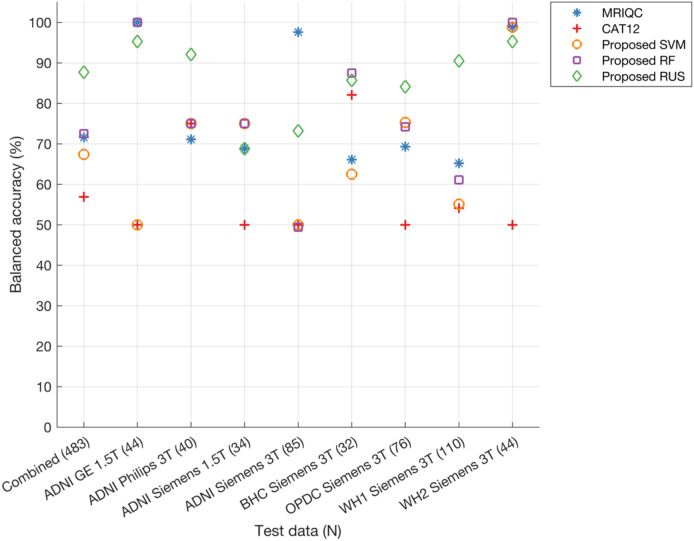
Balanced accuracy of proposed classifiers, MRIQC and CAT12 on combined and site-wise test data. The model’s performance on the test data is assessed across different (ranked) feature sizes (referSupplementary Fig. S2), with the displayed plot here representing only the best performance selected across these feature sizes. Number of samples in the test data are provided in brackets for each dataset (x-axis). Note that three sites (ADNI GE 3T, ADNI Philips 1.5T, and ADNI Siemens 2.9T) are not included in the figure due to the absence of samples in the reject class resulting in NaN values for balanced accuracies.

The optimal feature size selected for SVM (balanced accuracy = 67.4%) and RF was 50 (balanced accuracy = 72.5%), while that for RUS was 80 (balanced accuracy = 87.7%) (Fig. 5). On an average across different feature sizes for the combined test data, the proposed RUS classifier showed the highest balanced accuracy (85.2 ± 2.8%) as compared with SVM (62.8 ± 4.9%) and RF classifier (65.8 ± 3.7%) (refer to Supplementary Document for further details with confusion matrices (Supplementary Fig. S1) and performance at each feature size (Supplementary Fig. S2—site-wise performances,Supplementary Fig. S3—diagnosis-wise performances) for proposed classifiers and automated tools; additionally true positive, false positive rates, and balanced accuracies are reported in theSupplementary Excel filesheet named “Performance—Combined Test data”). The comparison of the best performance of the proposed classifiers with MRIQC and CAT12 showed that CAT12 (56.9%) gave the lowest balanced accuracy on the test data as compared with all classifiers, while MRIQC (71.6%) showed higher balanced accuracy than SVM but lower than RF and RUS classifiers. The higher performance of our proposed classifier shows the benefit of combining features from both MRIQC and CAT12. The results of further exploration of our best performing RUS classifier trained separately on MRIQC and CAT12 features, together with extensive variability analysis (on different splits of training and test data in 100 runs of cross-validation and on the final test data), are provided in theSupplementary Materials(Supplementary Analysis 1) (Varoquaux & Colliot, 2023).

When looking at the performance for each site separately in the test data (Fig. 5), the proposed classifiers showed higher balanced accuracies than CAT12 (except for BHC Siemens 3T where CAT12 showed higher balanced accuracy only when compared with SVM). We found that RUS achieved the highest balanced accuracies for 3 sites (ADNI Philips 3T, OPDC Siemens 3T and Whitehall 1 Siemens 3T sites). For other sites (ADNI GE 1.5T, ADNI Siemens 1.5T, BHC Siemens 3T, Whitehall 2 Siemens 3T), either MRIQC or RF showed the highest balanced accuracies, but RUS performance was also very close. For ADNI Siemens 3T site, MRIQC showed the highest balanced accuracy (97.6%), followed by RUS classifier (73.2%).

##### 3.3.1.1 Performance for patients and controls in test data

**Fig. 6.**
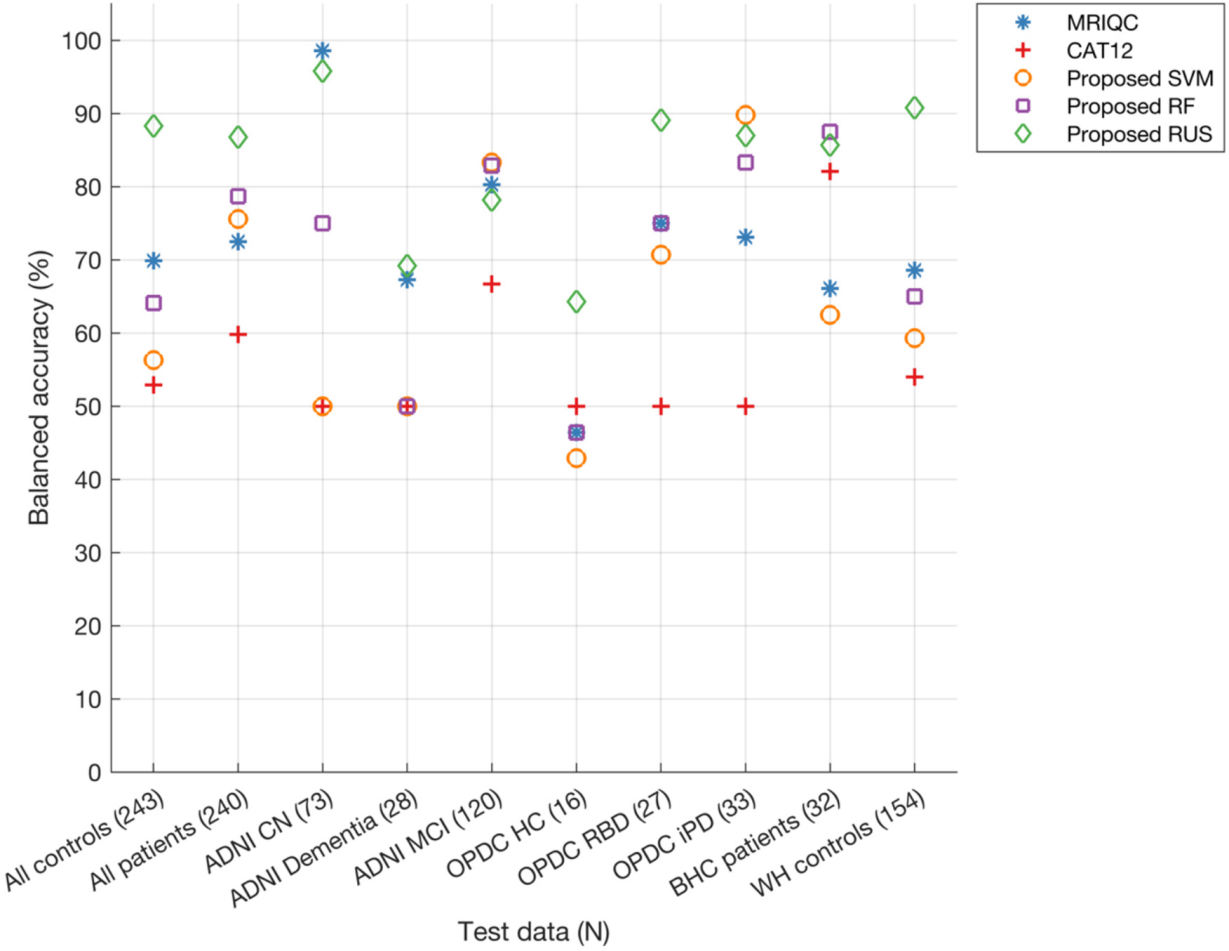
Balanced accuracy of proposed classifiers, MRIQC, and CAT12 analysed separately for scans from healthy individuals, patients, and each diagnostic sub-category within both healthy and patient groups in the test dataset. The model’s performance on the test data is assessed across different (ranked) feature sizes, with the displayed plot representing only the best performance selected across these feature sizes (referSupplementary Fig. S3). Number of samples in the test data are provided in brackets for each category (x-axis). Legend of diagnostic subgroups: CN = cognitively normal; HC = healthy controls; MCI = mild cognitive impairment; RBD = REM sleep behaviour disorder; iPD = idiopathic Parkinson’s disease.

Since our aim is to have a classifier that is suitable for clinical populations from different patient groups, we evaluated the performance separately for each disease group (For classifier performances on all groups refer to theSupplementary Excel filesheet named “Classifiers_performances”). When grouping scans in broad categories of patients (N = 240 in the test set, including AD, MCI, PD, RBD) and controls (N = 243 in the test set, generally cognitively unimpaired and without neurological conditions), the RUS classifier (balanced accuracy: patients = 86.8%, controls = 88.3%) achieved superior performance as compared with both SVM (balanced accuracy: patients = 75.6%, controls = 56.3%) and RF classifier (balanced accuracy: patients = 78.7%, controls = 64.1%). The RUS classifier performance was also higher than MRIQC (balanced accuracy: patients = 72.5%, controls = 69.9%) and CAT12 (balanced accuracy: patients = 59.8%, controls = 52.9%) (Fig. 6).

When looking at the performance for different diagnostic groups within ADNI, for the MCI group MRIQC, RF and SVM classifiers showed the highest balanced accuracy (>80%), while RUS accuracy was also very close to these classifiers (78%). The RUS classifier showed the highest balanced accuracy on the dementia group (69.2%) with MRIQC achieving 67.3%. On BHC data (memory clinic patients), the proposed SVM (87.5%) showed the highest balanced accuracy with the RUS classifier achieving 85.7%. When looking at the performance for different diagnostic groups within OPDC, for the RBD group, the RUS classifier showed the highest balanced accuracy (89.1%). For the iPD group, the proposed SVM and RUS classifiers showed the highest balanced accuracies (>85%). Upon comparing balanced accuracies across control groups from various datasets, the proposed RUS classifier demonstrated superiority for most datasets, achieving 64.3% for OPDC HC and 90.8% for Whitehall 2 controls. The only exception is the ADNI CN group, where the MRIQC classifier achieved the highest balanced accuracy at 98.6% with the RUS classifier performing close to MRIQC (95.8%).

##### 3.3.1.2 Artefact detection

Our classifiers effectively identified various artefacts present in the scans, including bias field, low contrast, motion artefacts, blurring, and incidental findings. All the classifiers predicted the unusable quality scans correctly (see example case inFig. 7a). Notably, we encountered cases where both MRIQC and CAT12 tools failed to make accurate predictions, while our classifiers correctly identified scans of reject quality (see example case inFig. 7b). Specifically, when RF and SVM classifiers, MRIQC and CAT12, inaccurately predicted reject quality scans, the RUS classifier made the correct prediction (see example cases inFig. 7c and d). Moreover, we noted instances where the RUS classifier misclassified some scans with accept quality into reject quality; however, the RF and SVM classifiers achieved accurate predictions in these cases (see example cases inFig. 7e and f).

**Fig. 7.**
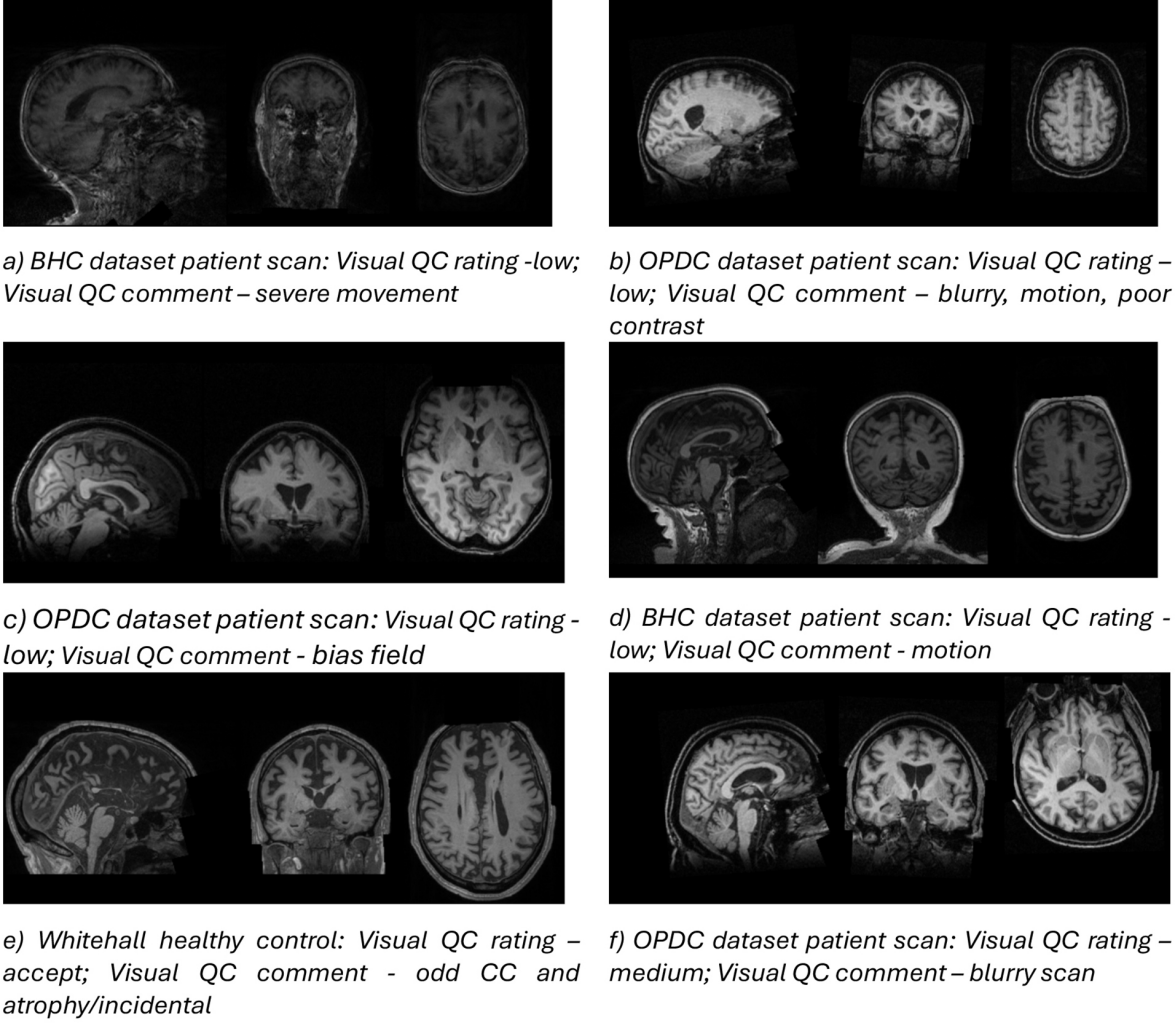
Example cases of quality prediction on test data. For each case, we additionally provide the visual QC report’s comment on the scans. The cases include (a) and (b) example scans which were correctly predicted into reject class by all the proposed classifiers, (c) and (d) example scans which were correctly predicted into reject class by the proposed RUS classifier, (e) and (f) example scans which were incorrectly predicted into reject quality by the proposed RUS classifier but correctly predicted into accept quality by RF and SVM classifiers.

##### 3.3.1.3 Feature importance

The feature ranking of the final model (combined data model) included features from both CAT12 and MRIQC in the top ranked features (referSupplementary Excel sheet“Feature ranks” to see feature ranks). The top 80 features (feature size showing the best balanced accuracy for the proposed RUS classifier) included 23 features (out of 36) from CAT12 [noise, contrast ratio, surface, and tissue measures] and 57 (out of 68) features from MRIQC [summary measures, noise measures, and tissue measures]. We selected the top 10 features (from 80 features) and plotted them to explore the distribution of these QC measures for each site in datasets (see KS density plots inFig. 8for different sites in ADNI andFig. 9for other sites). The plots reveal significant variations in the distribution of the top 10 features among different sites, highlighting technical variability despite the datasets originating from scanners of the same manufacturer. For instance, the disparity in feature distribution between the BHC dataset and others, despite all being acquired on 3T Siemens scanners, is evident (refer toFig. 9, panels b, c, d, f, i, j). Conversely, the distribution of features in the ADNI dataset suggests a more consistent pattern across various sites (refer toFig. 8, panels a to l).

**Fig. 8.**
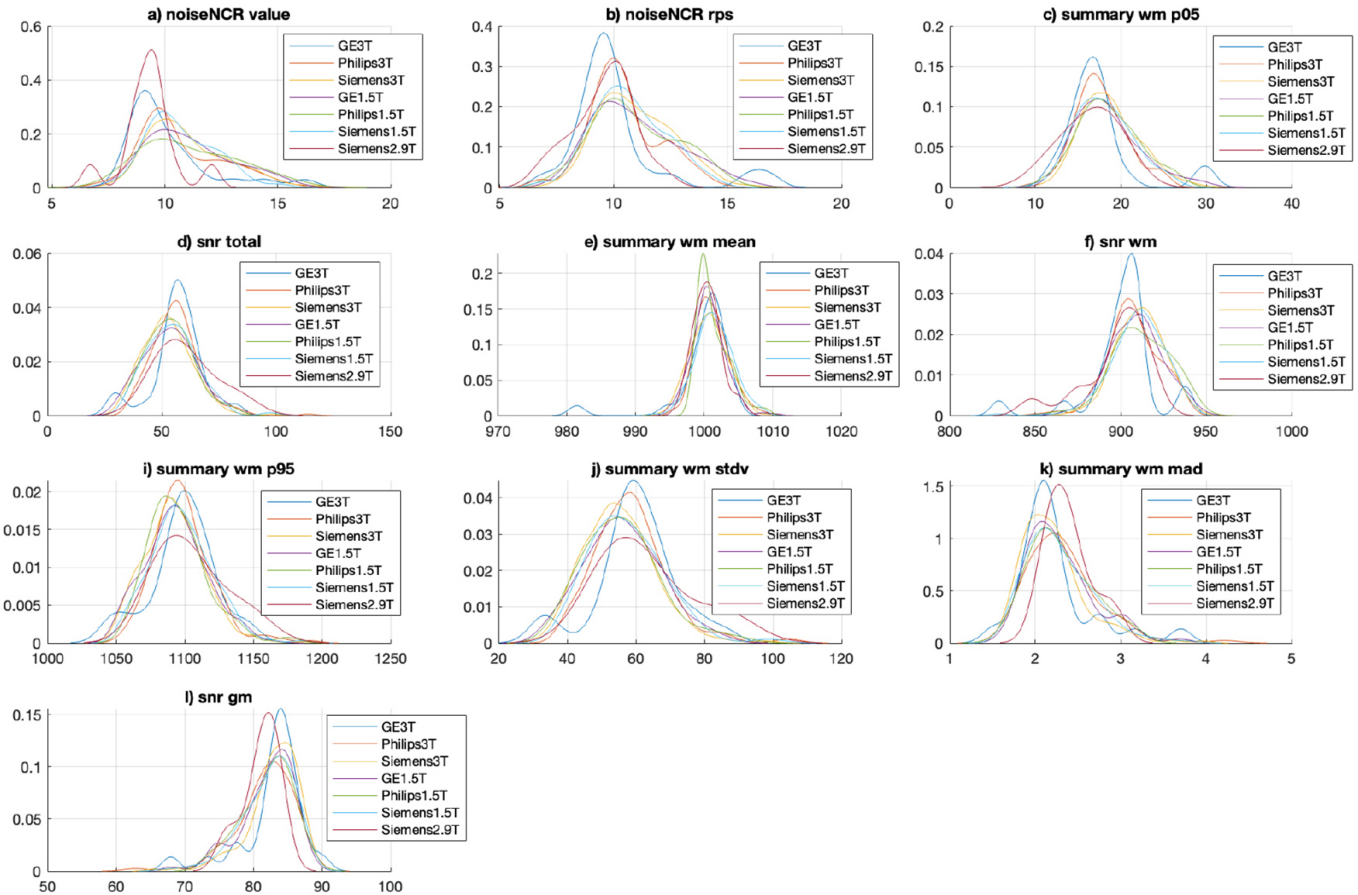
Kernel density plots showing the distribution of top 10 ranked features (panels a-l) in the final combined data model for sites within the ADNI dataset. For a description of the features, refer toTables 2and3.

**Fig. 9.**
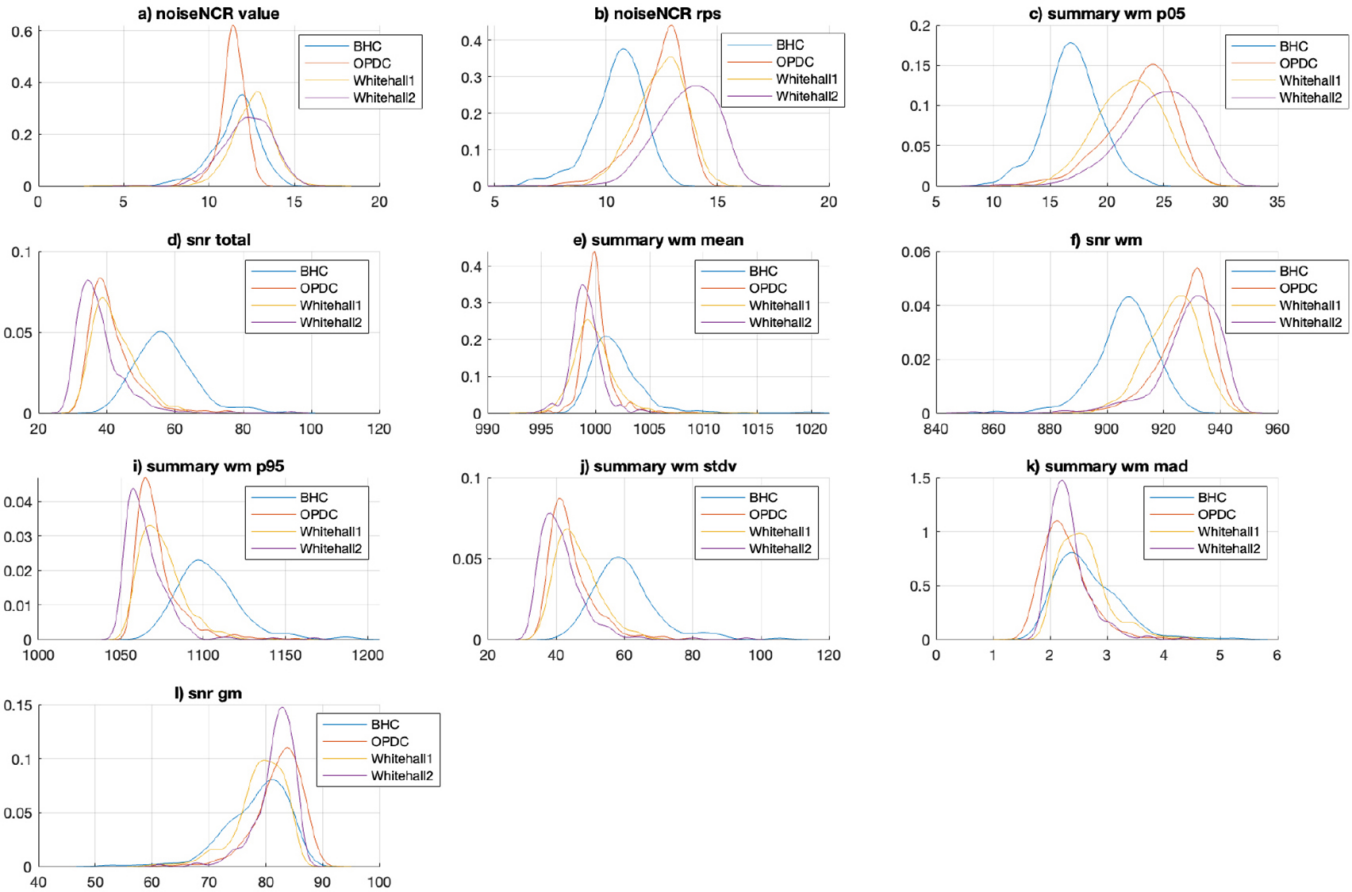
Kernel density plots showing the distribution of top 10 ranked features (panels a-l) in the final combined data model for BHC, OPDC, Whitehall1 and Whitehall2 datasets. For a description of the features, please refer toTables 2and3.

The statistical significance test (KS-test) conducted on the 80 features showed notable differences in the distribution between the pairs of sites. Details of KS-test on each pair of sites are reported in theSupplementary Excel file(sheet name—“kstest”). Briefly, significantly different distributions were observed for various features (>40% of 80 features) between the ADNI sites (Siemens—1.5T, 3T, GE—1.5T, 3T, Philips—1.5T, 3T) with the BHC, OPDC, and Whitehall sites. When comparing the distribution within ADNI sites, very few (<13% of 80 features) or none of the features showed significantly different distribution between the pairs of sites. Additionally, when comparing the distribution of features within non-ADNI sites (BHC, OPDC, Whitehall), many features (>67% of 80 features) showed significantly different distributions.

**Fig. 10.**
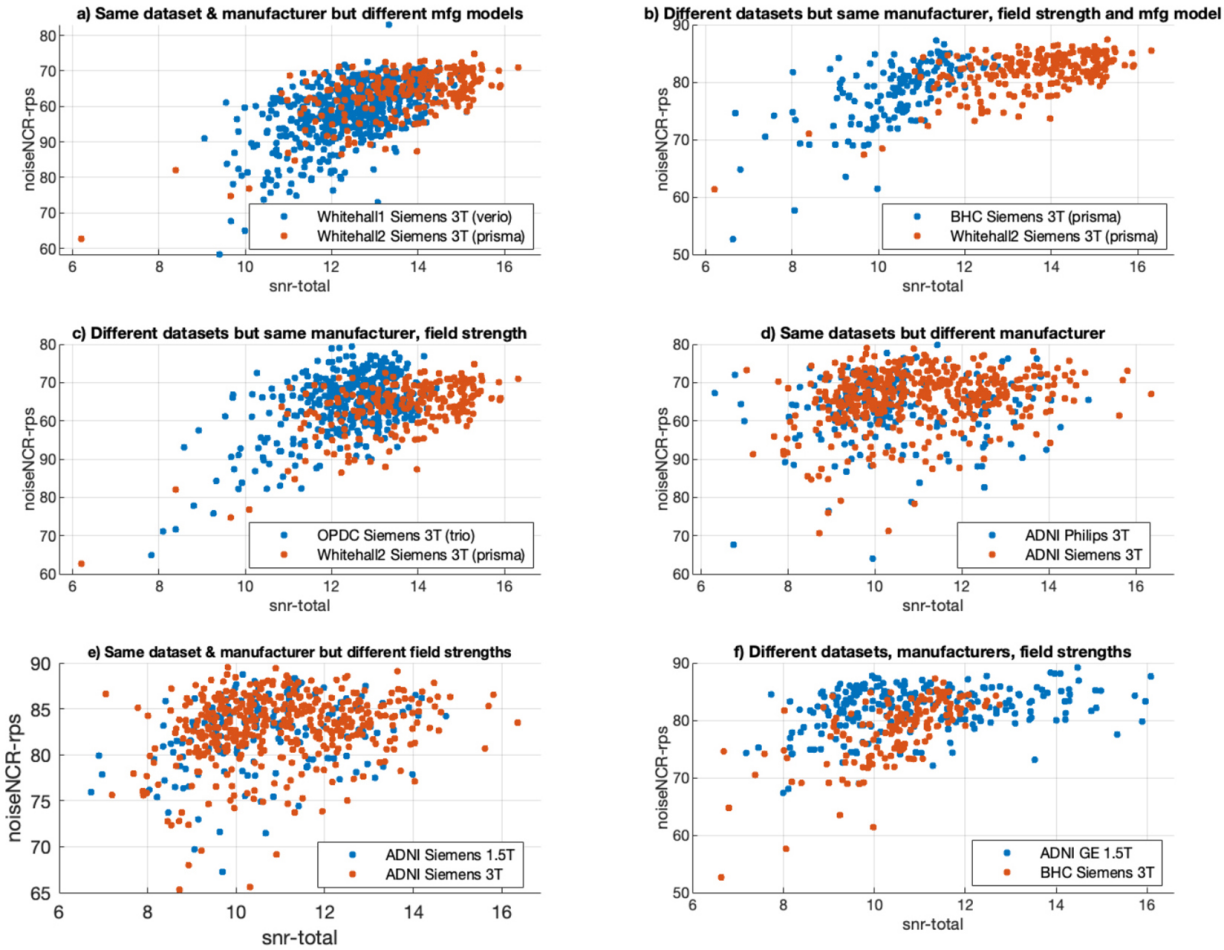
Scatter plots for two features snr-total from MRIQC on x-axis and noiseNCR-rps from CAT12 on y-axis showing different levels of overlap for different combinations of dataset, field strength and manufacturer: a) same dataset (Whitehall2), manufacturer (Siemens) and field strength (3T) but different scanner models; b) same scanner model (Siemens 3T Prisma) but different datasets; c) same manufacturer and field strength (Siemens 3T) but different datasets; d) same dataset (ADNI) and field strength (3T) but different manufacturers; e) same dataset (ADNI) and manufacturer (Siemens) but different field strengths; f) different datasets, manufacturers and field strength.

As an example,Figure 10presents scatter plots illustrating the relationship between two features: noiseNCR_rps (CAT12) and snr_total (MRIQC). These plots offer insights into the distribution patterns of these features across various scenarios. Noticeable clustering is observed between two different datasets (BHC from Siemens 3T and ADNI GE 1.5T,Fig. 10panel f), acquired from distinct scanner manufacturers and field strengths. However, there is no clustering within the sites of ADNI dataset irrespective of the difference in the scanner manufacturer (ADNI Philips 3T and ADNI Siemens 3T,Fig. 10panel d) and field strength (ADNI Siemens 1.5T and ADNI Siemens 3T,Fig. 10panel e). Another notable observation is the evident clustering observed between the BHC and Whitehall 1 datasets, despite both datasets being acquired using Siemens 3T Prisma scanners (Fig. 10panel b).

#### 3.3.2 Leave-one-site-out models

**Fig. 11.**
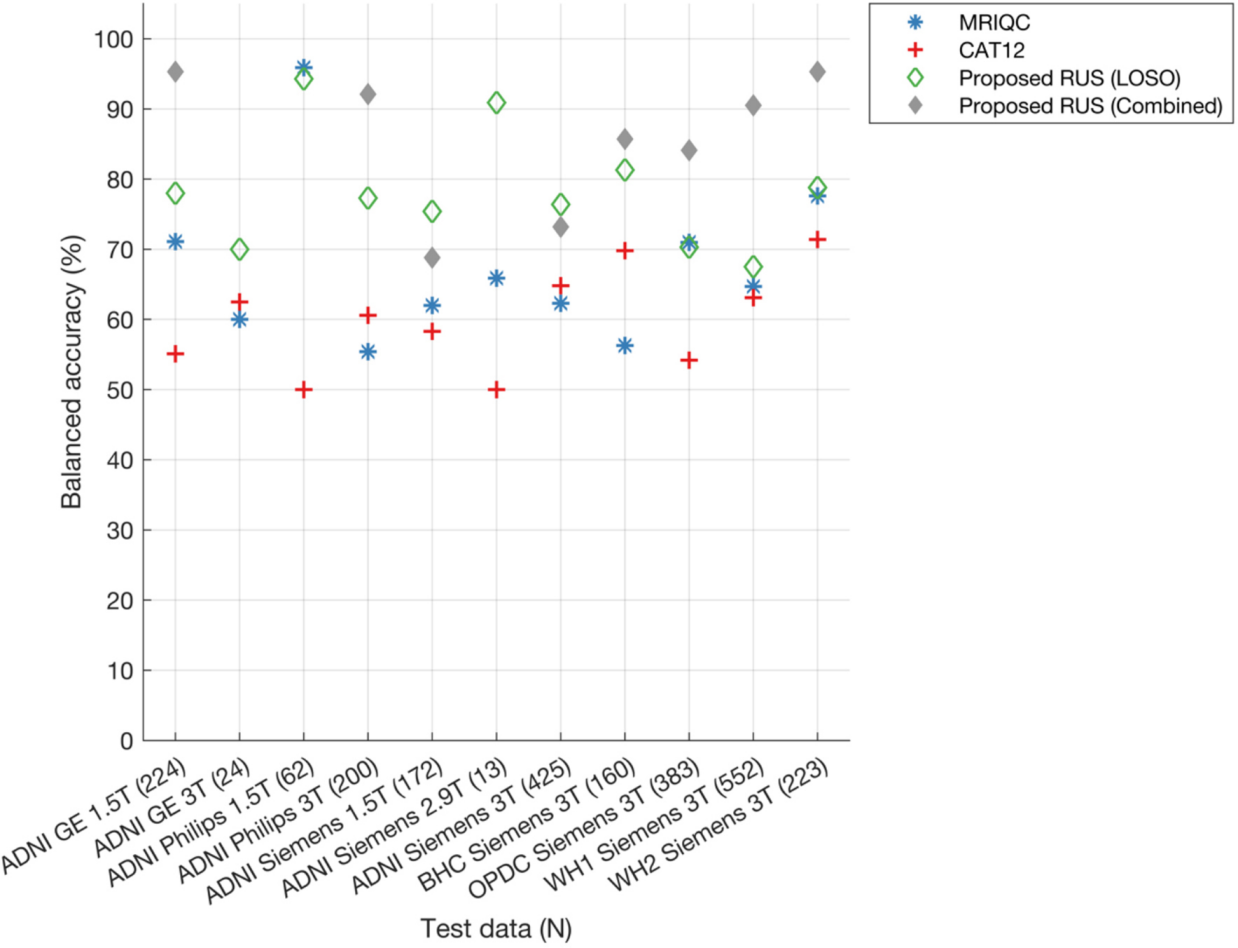
Balanced accuracy of MRIQC, CAT12, and the proposed RUS classifier for leave-one-site-out models. The model’s performance on the test data is assessed across different (ranked) feature sizes (referFig. S4in the Supplementary Document), with the displayed plot here representing only the best performance selected across these feature sizes. The total number of samples for each test site is provided in brackets (x-axis). For RUS classifier, each site was kept as test data and classifier was trained on remaining sites using the hyperparameters and feature ranking derived from combined data model (best model with 80 feature size). For reference, we also provide the balanced accuracy of RUS classifier for each site within the test data of the combined data model to see how well our classifier generalises to test data from different sites (diamond marker with grey). Note that balanced accuracies for the combined data model are not included for three sites (ADNI GE 3T, ADNI Philips 1.5T, and ADNI Siemens 2.9T) due to the absence of samples in the reject class of the test data (resulting in NaN values for balanced accuracies).

From the results on the combined model, the RUS classifier gave the best performance and was used for further experiments. The proposed RUS classifier achieved the highest balanced accuracy (78.2 ± 8.3%) across test sites as compared with MRIQC (67.5 ± 11.5%) and CAT12 (60 ± 7.2%) (Fig. 11) (Also referSupplementary Materials Fig. S4for performance at each test site across feature sizes). When comparing the balanced accuracy for each site, the proposed RUS classifier consistently performed better than MRIQC, except at three sites where the performance values were similar or close. These sites include ADNI Philips 1.5T (1.6% difference, RUS lower), OPDC Siemens 3T (0.6% difference, RUS lower), and Whitehall 2 Siemens 3T (1.1% difference, RUS higher). As expected, the balanced accuracy for individual sites in leave-one-site-out models tended to be lower than the results from the combined data model (average across sites = 85.6 ± 10%, displayed for reference inFig. 11), due to fewer samples available in the test data and the presence of site-specific data in the training set for the combined data model.

Additionally refer to the results provided in theSupplementary Materials(Supplementary Analysis 2) when leave-one-site-out models were trained from scratch (that means without inheriting the model parameters from the combined data model).

#### 3.3.3 Exploratory models

**Fig. 12.**
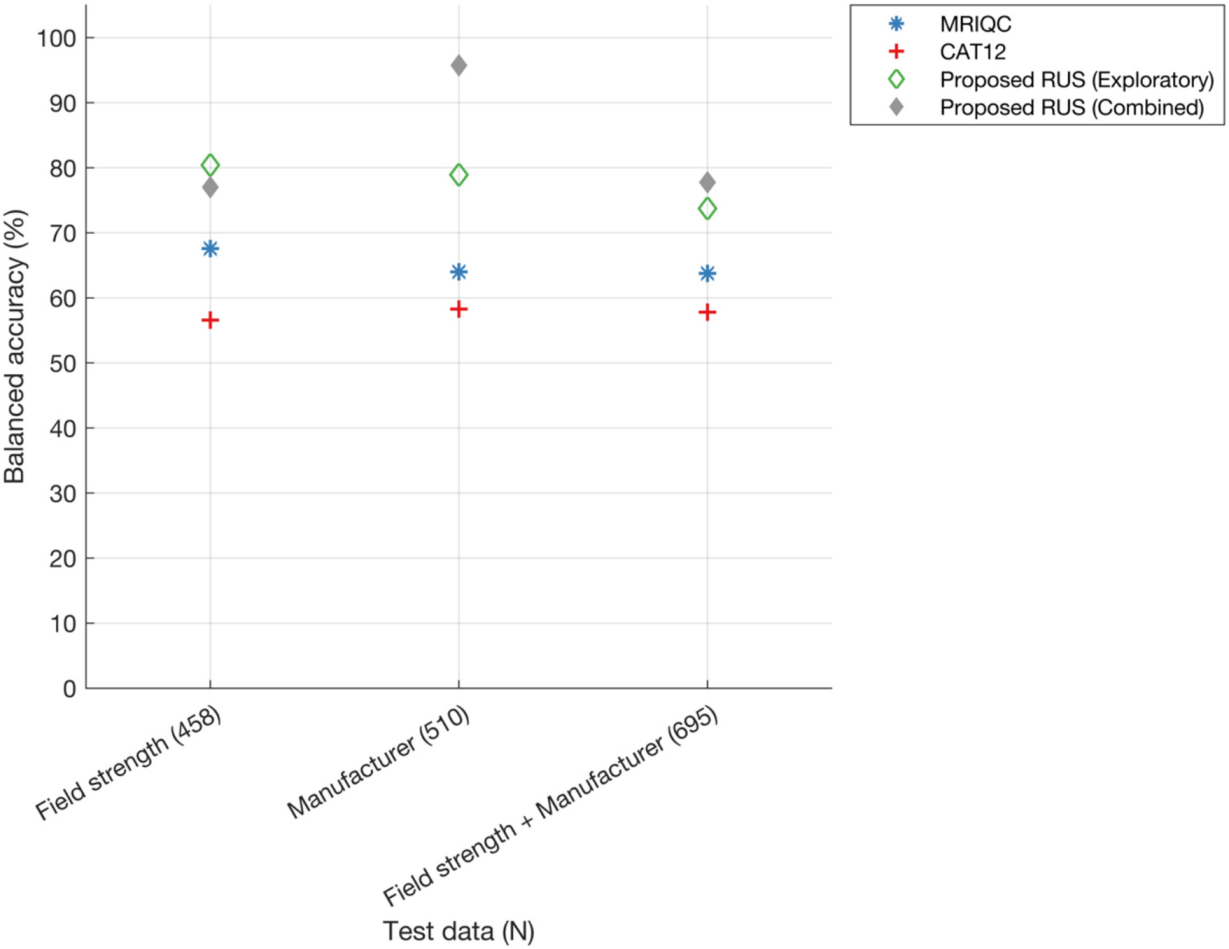
Balanced accuracy of MRIQC, CAT12, and the proposed RUS classifier for exploratory models on field strengths and manufacturers. The model’s performance on the test data is assessed across different (ranked) feature sizes (referFig. S5in the Supplementary Document), with the displayed plot here representing only the best performance selected across these feature sizes. The total number of samples for each test site is provided in brackets (x-axis). Field strength: performance of models trained on 3T scanners data (Siemens, Philips, GE) and tested on 1.5T (Siemens, Philips, GE) and 2.9T (Siemens) scanners data; manufacturer: performance of models trained on Siemens (1.5T, 2.9T, 3T) data and tested on Philips (1.5T, 3T) and GE (1.5T, 3T) data; manufacturer and field strength: performance of models trained on Siemens 3T data and tested on Siemens (1.5T, 2.9T), Philips (1.5T, 3T), and GE (1.5T, 3T) data. Additionally, the balanced accuracy of the RUS classifier within the test data for the combined data model for each scenario is presented for reference (diamond marker with grey).

For all three exploratory models on field strengths and manufacturers, the proposed RUS classifier consistently showed the highest balanced accuracies (73.8%–80.4%) compared with MRIQC (63.8%–67.9%) and CAT12 (56.6%–58.3%) (Fig. 12) (Also referSupplementary Materials Fig. S5for performance across feature sizes). Additionally, when comparing performance across exploratory models, the model trained on 3T scanners and tested on 1.5T scanners data showed higher balanced accuracy (80.4%) than the other two cases (manufacturer = 78.9%, field strength and manufacturer = 73.8%), probably due to the higher number of training samples (seeTable 7). The “manufacturer” model trained with Siemens data (1.5T, 2.9T, 3T) showed 84% balanced accuracy on Philips scanner data (1.5T, 3T) and 75% balanced accuracy on GE scanner data (1.5T, 3T). The model trained with 3T Siemens data (field strength + manufacturer) showed 72.4% balanced accuracy on test data from Siemens scanner (1.5T, 2.9T), 73.3% balanced accuracy on test data from GE scanner (1.5T, 3T), and 76.6% balanced accuracy on test data from Philips scanner (1.5T, 3T). Also, in this case, the performance on test data from the combined model for each of the three models (reported for reference inFig. 12) showed higher balanced accuracies (except field strength exploratory model which achieved 3.4% higher accuracy).

Additionally refer to the results provided in theSupplementary Materials(Supplementary Analysis 2) when exploratory models were trained from scratch (that means without inheriting the model parameters from the combined data model).

**Fig. 13.**
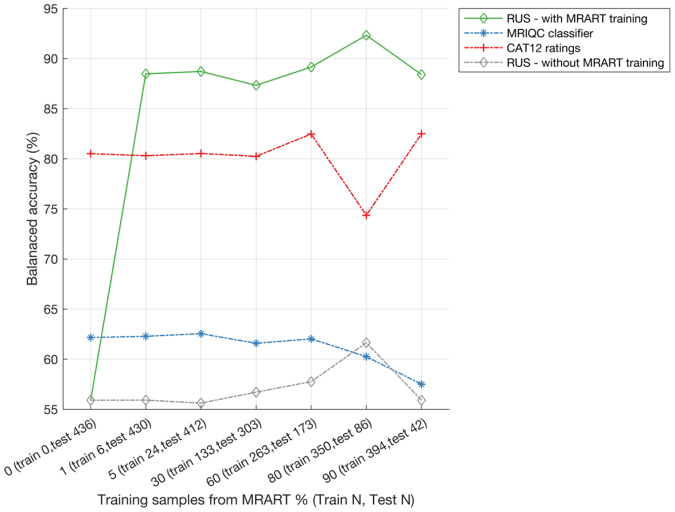
Balanced accuracy of MRIQC, CAT12, and the proposed RUS classifier for exploratory models on the MR-ART dataset. We tested the option of applying our combined data model without adding MR-ART samples (RUS—without MRART training) and after retraining including a proportion of MR-ART images. X-axis indicates the % training samples used in addition to main training data (*N*= 1955) and total number of training and test samples from MR-ART (details inTable 8). Feature ranking, feature size, and hyperparameters were taken from the combined data model.

Regarding the exploratory models on the MR-ART dataset, when applying the RUS classifier to this new dataset of young healthy individuals, it showed lower balanced accuracy than MRIQC and CAT12 (MRIQC 62.2%, CAT12 80.5%, RUS 55.9%) but outperformed them in specificity (ability to correctly predict reject-quality scans—MRIQC 24.7%, CAT12 63.1%, RUS 75.3%). When incorporating labelled MR-ART samples into the training process (adding MR-ART samples to the original training data), the proposed RUS consistently outperformed MRIQC and CAT12 tools (RUS balanced accuracy 88.5%), even when including just 1% (6 scans) of MR-ART data into the training (Fig. 13). Refer toSupplementary Materials(Supplementary Analysis 3) for more details on these results (Supplementary Fig. S15for sensitivity, specificity, and balanced accuracy plots,Supplementary Table S4for details on the performance comparison after adding before and after adding MR-ART samples to the original training data).

## 4 Discussion

In this study, we investigated approaches for automated quality control of T1w brain scans for ageing and clinical research datasets acquired from multiple sites. The existing tools assessed in this study, MRIQC and CAT12, offer a broad array of quality metrics both from raw and processed images. We observed that some of the metrics are common between the tools, either assessing the same measures or highly correlated measures, while others are unique (i.e., not significantly correlated to measures from the other tool). When looking at the agreement in the accept or reject ratings between these tools and with visual QC, we found high variability across datasets and only a “fair” agreement (McHugh, 2012) on the overall dataset (Kappa coefficient < 0.3), suggesting that these tools might not be suitable for highly heterogeneous clinical research datasets, and in the absence of visual QC (i.e., ground truth), the selection of the QC tool can significantly influence the decision on whether or not to include a specific scan in the analysis pipeline. We observed enhanced agreement between visual QC and these tools after modifying the acceptance threshold. Nevertheless, these enhancements varied across different datasets and on the overall dataset, this improvement was only observed with one of the tools (CAT12; Kappa coefficient improved from 0.28 to 0.35 with visual QC), indicating that the adjusted thresholds may not be suitable for all clinical research datasets. We then proposed a QC prediction approach by combining the quality measures from the automated tools to create a new classifier. The proposed RUS classifier exhibited higher performance than SVM and RF and good generalisability of prediction on the test datasets from diverse sites, scanner manufacturers, and field strengths (balanced accuracy 87.7% on combined test data; average balanced accuracy 78 ± 8.3% on 11 test sites; average balanced accuracy 77.7 ± 3.5% on exploratory models testing generalisability to field strengths and manufacturers). Further, we observed that extracted QC metrics did not only enhance prediction performance but also effectively captured the variability across different scanners and datasets. With these insights, our future work will focus on streamlining the extraction of these measures directly from clinical scans, bypassing the need for extensive preprocessing pipelines.

The proposed RUS classifier combining features from MRIQC and CAT12 outperformed the existing tools suggesting that MRIQC and CAT12 features offer complementary information. This is evident from the feature ranking, where the selected features at the top originated from both tools. To further disentangle the benefit of combining features from the use of a different classifier, we re-trained the RUS model with MRIQC and CAT12 separately. This additional analysis, detailed in theSupplementary Material(Supplementary Analysis 1), revealed that the combination of features still offers the best performance. Additionally, we explored the distribution of the quality measures that significantly contributed to the high performance (top ranked features) and observed that certain measures effectively captured variations across datasets (even for datasets acquired using scanners from the same field strength and manufacturer, for example, BHC and Whitehall 2 datasets both acquired on Siemens 3T Prisma scanners). This highlights the complex technical differences among datasets, which might be influenced not solely by scanner manufacturer or field strength, but also by other factors (e.g., acquisition parameters, number of channels in head coil, cohort characteristics such as age, sex, and diagnosis). These quality measures, when used in the context of harmonisation techniques such as Neuroharmony (Garcia-Dias et al., 2020), could be instrumental in mitigating site-related effects in studies involving data from multiple sites.

Due to the highly curated nature of the datasets, resulting in a significant imbalance between accept and reject labels, we focused on optimising balanced accuracy rather than overall accuracy. We also implemented multiple iterations of nested cross-validation (total 100) to iteratively validate and train our model on different samples. The use of the RUS classifier effectively addressed the issue of class imbalance by implementing under-sampling on the majority class (accept-labelled scans) to match it with the minority class (reject-labelled scans) during the training phase. This is clearly demonstrated by the improved specificity in predicting reject class labels, resulting in a notable enhancement in the balanced accuracy of RUS prediction when compared with RF, SVM, and other automated tools.

The RUS classifier achieved comparable performance on data from patients and controls (balanced accuracy of 86.8% and 88.3%, respectively). We observed differences in performance across diagnostic subgroups, but while for ADNI the performance was lower in dementia than in controls, in OPDC the performance was lower for controls than in PD patients. This suggests that while diagnostic status of scans could affect results, the results may also be influenced by the total number of samples and number of scans in the reject class within each subgroup, making it difficult to perform a fair direct comparison across subgroups. We used a similar number of samples from both classes (accept and reject) in the training and test datasets for patients and controls, but not necessarily balanced within subgroups, due to the differences highlighted above. The additional validation of our combined data model on different test sites (leave-one-site-out models) and test data from various scanners and field strengths (exploratory models) demonstrate that our model generalises well according to these characteristics. However, when testing its generalisability on a new dataset of younger and healthier individuals with high levels of artefacts (MR-ART), we observed a reduction in performance below MRIQC and CAT12 (which had demonstrated poor performance on our ageing and clinical datasets), despite the fact that this dataset was acquired on a Siemens 3T scanner. Adding a small number of samples from the new dataset (1%, 6 scans) increased the performance to over 88.5% balanced accuracy. This indicates that, like other existing classifiers, achieving a consistently high performance on all datasets and populations is challenging. However, our specific goal was to address a current limitation of commonly used tools, which was the ability to offer high performance on clinical research datasets from ageing and dementia populations. Our analyses showed the benefit of using a wide set of features for the classifier, an algorithm that deals with the imbalance of accept and reject class (common scenario in research datasets) and proved that the performance on a new dataset can be significantly improved by adding a small number of labelled samples without the need to re-optimise hyperparameters.

The proposed approach also has the limitation of needing extensive pre-processing, such as segmentation, to extract certain QC metrics from the automated tools utilised in this approach. These processing steps are more prone to errors when applied to clinical data, potentially causing misclassifications if applied to routine scans. Nevertheless, having identified quality metrics that assist in assessing image quality within a clinical population through this approach, future work will focus on developing methods to extract these quality metrics from images more directly and robustly, extending the use of our approach beyond research datasets to routine clinical scans. A limitation of this study is that all T1w scans were acquired using gradient echo-based MPRAGE sequences. No fast spin echo-based scans were included in the training or evaluation of our QC classifier. Since these sequences may differ in contrast, artefact susceptibility, and noise properties, the classifier’s performance on such images remains untested. Future work should assess the model’s robustness across different T1w acquisition protocols. Another limitation arises from the use of defaced T1w scans which involves the removal of facial features to protect individuals’ privacy. This step modifies the image, potentially altering the characteristics used for quality control. Recent studies have demonstrated that that defacing can have some detrimental impact on the downstream image analysis (Bhalerao et al., 2022;Provins et al., 2023;Rubbert et al., 2022). While this issue remains an ongoing concern within the neuroimaging community, we decided to use defaced images as this is currently the best practice for sharing datasets and our goal was to develop a QC approach able to work on multiple datasets, likely aggregated from different sources on a data sharing and analysis platform, like the DPUK portal. For consistency, we applied the same defacing method (fsl_deface) across all datasets whenever possible, but MR-ART dataset was shared using pydeface, which may have contributed to the observed difference in performance. Another constraint stems from the fact that the visual QC was performed by different raters, as we relied on visual QC ratings provided by the dataset owners. From the descriptions provided by the data owners, the criteria used are comparable, however, different raters may have had different subjective threshold for quality control, which could have impacted the results. For example, in the MR-ART dataset, bad quality images were those considered unusable for clinical diagnostics, which is a potentially more lenient threshold than what was used for a research scan (i.e., an image could still be reported visually, but be too corrupted to generate reliable measures when analysed with automated tools). This could contribute to explain why our classifier rated more MR-ART scans in the reject class. However, potential differences in ratings reflect the real-world scenario of combining datasets from different sources, hence the decision to use labels from cohort owners. Despite the classifier’s ability to predict quality across the diverse ageing and neurodegenerative datasets used in this study, we acknowledge that applying it to entirely new datasets—varying in population, age, or disease type, etc.—could require adding examples from the new datasets to reach satisfactory performance. Additionally, our classifier is optimised to minimise false negatives (i.e., poor-quality subjects flagged as good) at the expense of a higher rate of false positives (i.e., accept-quality scans flagged as bad), which would require manual inspection (criteria similar to the UK Biobank pipeline’s QC tool (Alfaro-Almagro et al., 2018)).

Since our primary goal was to develop a classifier using existing datasets available for sharing, one of the challenges we encountered was the limited availability of poor-quality scans (reject class). This is because shared datasets often are already highly curated. Datasets such as MR-ART contain more reject scans but are inevitably less representative of a real-world clinical study, as motion artefacts were introduced by design and all participants were healthy. We highly recommend to the community to also share the poor-quality data from research studies on patient populations, which can enhance the generalisability of classification on new datasets and ultimately to real-world clinical scans. Our classifiers and code are publicly available and have been designed with flexibility in mind, allowing for straightforward integration and retraining with new datasets as they become available in the future. Another strategy to address this issue involves leveraging synthetic image generation techniques. For instance, new datasets can be created by artificially introducing image artefacts into MRI scans derived from real-world data, thereby augmenting the sample size within the reject class (Ravi et al., 2024). However, the challenge is to create images that simulate realistic artefacts (Giuffrè & Shung, 2023). Another potential future direction would be to test the inclusion of more QC features (such as from FreeSurfer tool (Dale et al., 1999) or UK biobank neuroimaging pipeline (Alfaro-Almagro et al., 2018)) in our classification framework to test whether they result in increased performance (without significantly increasing the computational load) and/or further improve the generalisation of our classifier to new datasets. Our model is available to the community, and we plan to extend similar framework to test the quality of other MRI modalities.

## 5 Conclusion

We proposed a classification model for quality assessment of T1-weighted scans of clinical research datasets originating from diverse scanners, acquisition protocols, spanning an elderly age range and including different neurodegenerative conditions. Our approach involved combining the quality measures derived from automated tools, yielding promising performance, particularly when dealing with heterogeneous datasets from ageing and diseased cohorts. The code is readily available, and we will also share the QC metrics, trained classifiers, and outputs of this work through the DPUK data portal. This resource will serve as an asset for further exploration and robust QC of T1w scans across datasets, promoting comprehensive and reliable image quality assessment in future studies.

## Supplementary Material

Supplementary Material

QC_paper_supplementary_R2

## Data Availability

Code, trained models, andSupplementary Materialsare available here: (https://git.fmrib.ox.ac.uk/mcz502/qc-paper). Access to ADNI data is available to researchers upon request and approval of a data usage agreement (https://adni.loni.usc.edu/). Details on how to request access to the data can be found at (https://adni.loni.usc.edu/data-samples/adni-data/neuroimaging/mri/mri-image-data-sets/). Other datasets used in this study can be accessed through the submission of an application through the DPUK data portal (https://portal.dementiasplatform.uk/Apply). MR-ART dataset and its ground truth labels are openly available to download on OpenNeuro (https://openneuro.org/datasets/ds004173/versions/1.0.2). Access to manual QC labels: For the ADNI dataset, these labels are included with data access. For the other three datasets, they can be obtained from the dataset owners or the authors upon request, provided that access to the datasets is successfully approved.
