# Supplementary material for: Automated quality control of T1-weighted brain MRI scans for clinical research datasets: methods comparison and design of a quality prediction classifier
Source: Imaging Neurosci (Camb). 2025 May 28;3:IMAG.a.4. doi: 10.1162/IMAG.a.4 (PMC12319838; doi:10.1162/IMAG.a.4)
Supplement: Supplementary Material [file imag.a.4_supp1.pdf]

Automated quality control of T1-weighted brain MRI scans for clinical research datasets: methods comparison and design of a quality prediction classifier

- Supplementary materials

Supplementary materials

Training and test data split for combined data models 2
Performance of classifiers on combined test data 3
Performance of RUS classifier for leave-one-site-out models 6
Performance of RUS classifier for the exploratory models 7
Supplementary analysis 1 8
Supplementary analysis 2 14
Supplementary analysis 3 20

Automated quality control of T1-weighted brain MRI scans for clinical research datasets: methods comparison and design of a quality prediction classifier

- Supplementary materials

Training and test data split for combined data models

Table S1. Training and test data split counts for each dataset, diagnosis group, scanner field strength and manufacturer

Dataset	*Diagnosis*	*Sub-group*	*Field Strength(T)*	*Scanner Manuf*	*Train Total*	*Train Accept*	*Train Reject*	*Test Total*	*Test Accept*	*Test Reject*
ADNI	control	CN	1.5	GE	51	51	0	17	16	1
ADNI	control	CN	1.5	PHILIPS	9	9	0	5	5	0
ADNI	control	CN	1.5	SIEMENS	46	42	4	8	8	0
ADNI	control	CN	2.9	SIEMENS	2	1	1	0	0	0
ADNI	control	CN	3	GE	5	3	2	2	2	0
ADNI	control	CN	3	PHILIPS	51	47	4	9	9	0
ADNI	control	CN	3	SIEMENS	101	99	2	32	31	1
ADNI	patient	Dementia	1.5	GE	32	26	6	6	6	0
ADNI	patient	Dementia	1.5	PHILIPS	8	7	1	1	1	0
ADNI	patient	Dementia	1.5	SIEMENS	33	29	4	8	8	0
ADNI	patient	Dementia	2.9	SIEMENS	2	1	1	0	0	0
ADNI	patient	Dementia	3	GE	2	1	1	0	0	0
ADNI	patient	Dementia	3	PHILIPS	25	25	0	5	4	1
ADNI	patient	Dementia	3	SIEMENS	62	59	3	8	7	1
ADNI	patient	MCI	1.5	GE	97	95	2	21	21	0
ADNI	patient	MCI	1.5	PHILIPS	33	33	0	6	6	0
ADNI	patient	MCI	1.5	SIEMENS	59	57	2	18	16	2
ADNI	patient	MCI	2.9	SIEMENS	7	7	0	2	2	0
ADNI	patient	MCI	3	GE	13	12	1	2	2	0
ADNI	patient	MCI	3	PHILIPS	84	81	3	26	25	1
ADNI	patient	MCI	3	SIEMENS	177	174	3	45	45	0
BHC	patient	-	3	SIEMENS	128	116	12	32	28	4
OPDC	control	HC	3	SIEMENS	74	66	8	16	14	2
OPDC	patient	RBD	3	SIEMENS	97	81	16	27	23	4
OPDC	patient	iPD	3	SIEMENS	135	113	22	33	27	6
OPDC	unknown	unknown	3	SIEMENS	1	0	1	0	0	0
Whitehall1	control	control	3	SIEMENS	442	407	35	110	101	9
Whitehall2	control	control	3	SIEMENS	179	173	6	44	43	1
				Total	1955	1815	140	483	450	33

Performance of classifiers on combined test data

The confusion matrix for each classifier along with MRIQC and CAT12 is shown in ***Figure S1***. The proposed RUS classifier showed the highest true negatives (reject labels correctly classified as reject) and the lowest false positives (reject labels wrongly classified as accept) as compared to all the other classifiers. The RF classifier showed the highest true positives (accept labels correctly classified as accept) and the lowest false negatives (accept labels wrongly classified as reject) as compared to all the other classifiers.

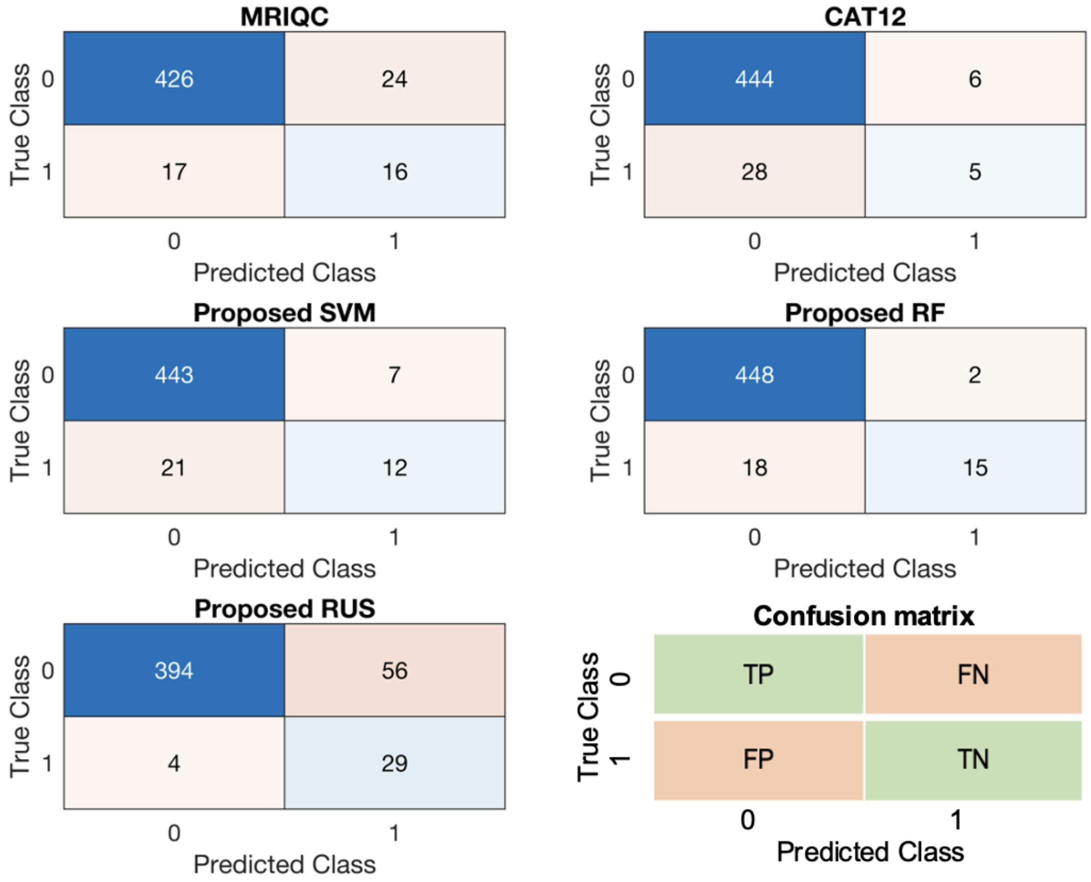

Figure S1. Confusion matrices showing total number of true positives, true negatives, false positives, and false negatives in the test data for MRIQC, CAT12 and proposed classifiers. Class 0 represents the accept class (positive), while class 1 represents the reject class (negative).

Automated quality control of T1-weighted brain MRI scans for clinical research datasets: methods comparison and design of a quality prediction classifier

- Supplementary materials

The optimal feature size selected for SVM (balanced accuracy - 67.4%) and RF was 50 (balanced accuracy -72.5%), while for RUS was 80 (balanced accuracy - 87.7%)

Site-wise plots across ranked feature sizes

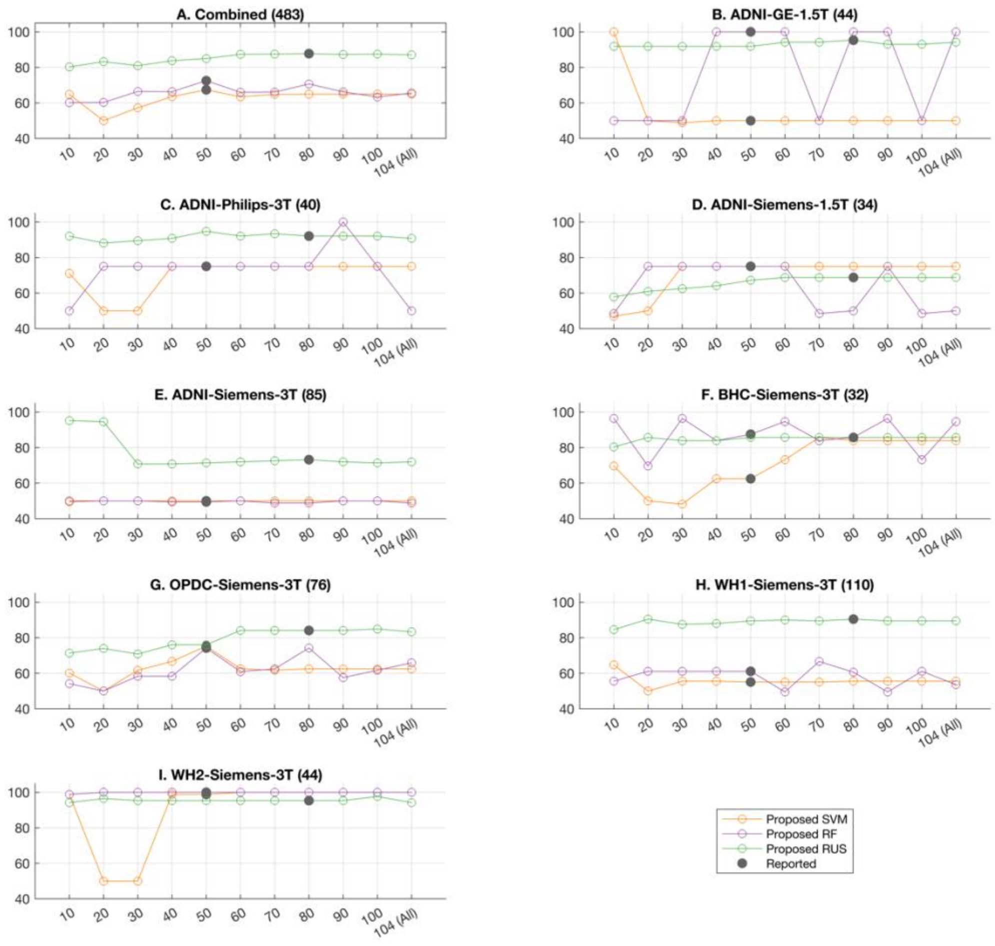

Figure S2. Balanced accuracies across feature sizes for combined data model tested on the combined test data (panel A) and split into sites (Panels B-I). On the combined data model, the best performance was achieved using the top 50 ranked features for SVM and RF, while RUS performed best with the top 80 ranked features (marked with a grey circle on the plot and displayed in figure 5 in the main text). Panels B-I show that the selected feature size (grey circle) gives good balanced accuracy in all sites, although not necessarily the best performance. We fixed the feature size based on the results on the combined data model to compare performance on the different sites in Figure 5.

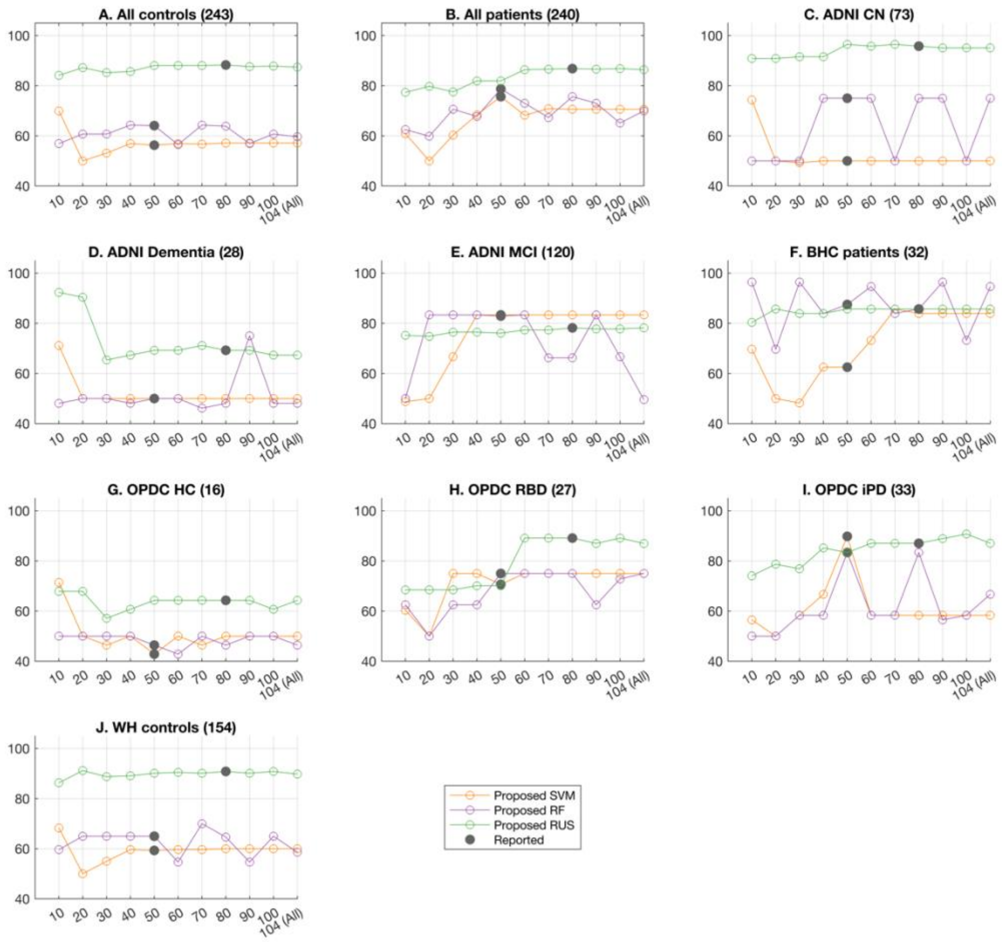

Figure S3. Balanced accuracies across feature sizes for combined data model split into controls (Panel A), patients (Panel B) and subgroups (Panels C-J). On the combined data model, the best performance was achieved using the top 50 ranked features for SVM and RF, while RUS performed best with the top 80 ranked features (marked with a grey circle on the plot and displayed in figure 5 in the main text). Panels A-J show that the selected feature size (grey circle) gives good balanced accuracy in all groups, although not necessarily the best performance. We fixed the feature size based on the results on the combined data model to compare performance on the different sites in Figure 5.

Automated quality control of T1-weighted brain MRI scans for clinical research datasets: methods comparison and design of a quality prediction classifier

- Supplementary materials

Performance of RUS classifier for leave-one-site-out models

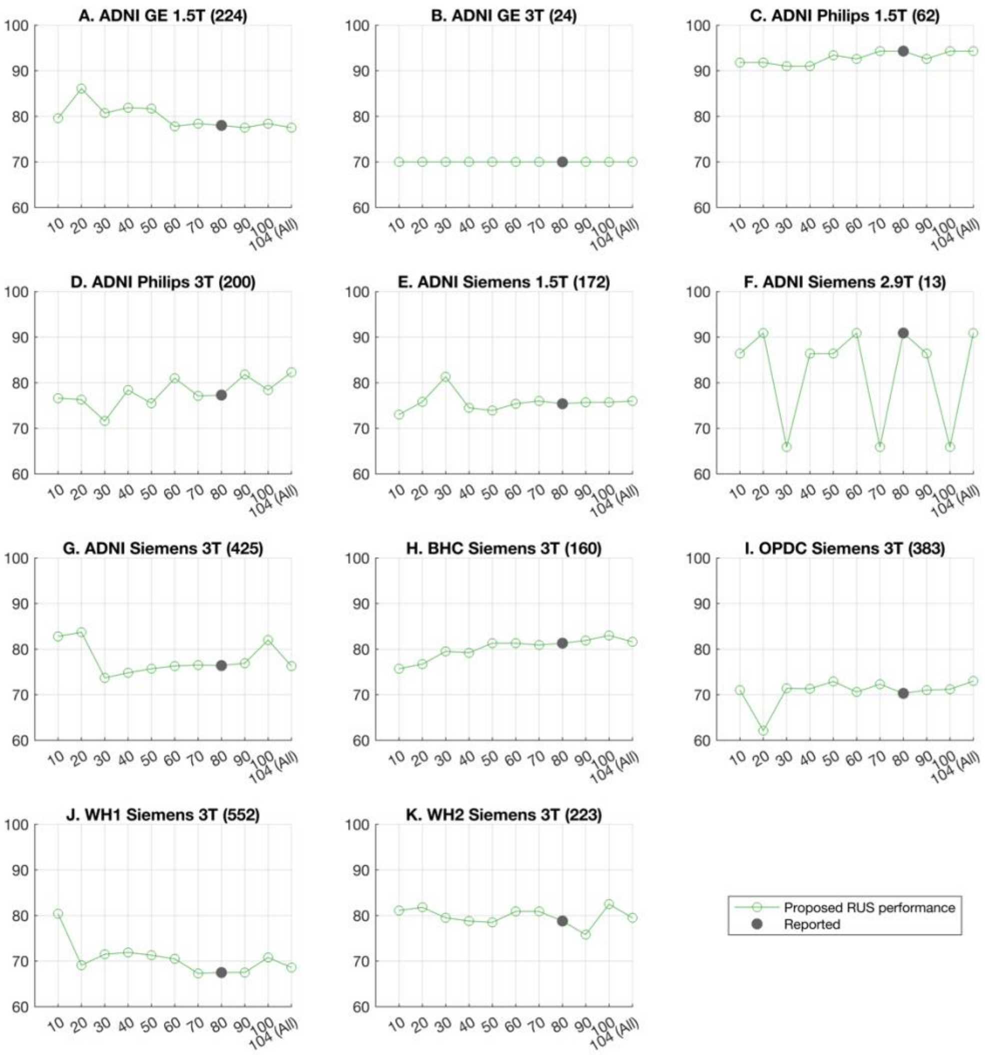

Figure S4. Balanced accuracy of proposed RUS classifier across different feature sizes for leave-one-site-out models. Panels A-K show that the selected feature size (grey circle) gives good balanced accuracy in all groups, although not necessarily the best performance. We fixed the feature size based on the results on the combined data model to compare performance on the different sites in Figure 5.

Performance of RUS classifier for the exploratory models

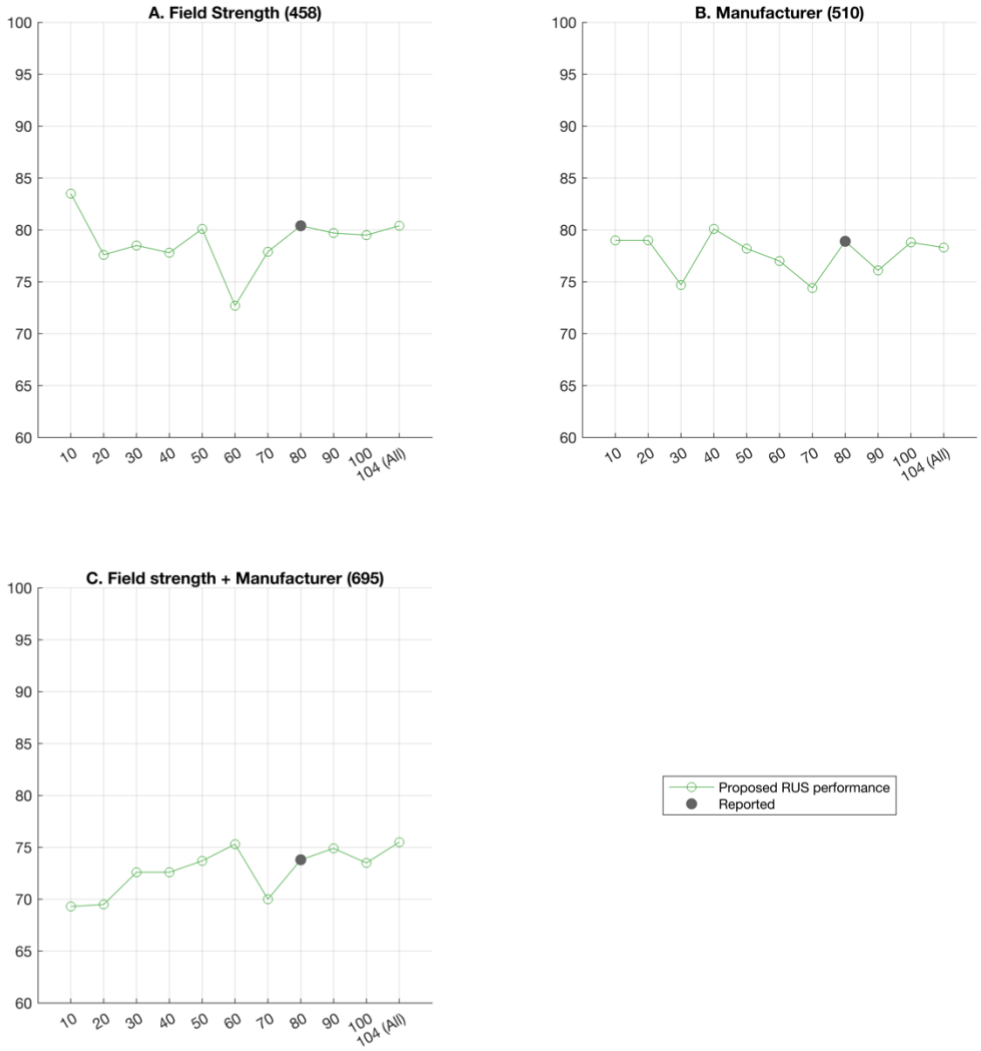

Figure S5. Balanced accuracy of proposed RUS classifier across different feature sizes for exploratory models. Panels A-C show that the selected feature size (grey circle) gives good balanced accuracy in all groups, although not necessarily the best performance. We fixed the feature size based on the results on the combined data model to compare performance on the different sites in Figure 5.

Automated quality control of T1-weighted brain MRI scans for clinical research datasets: methods comparison and design of a quality prediction classifier

- Supplementary materials

Supplementary analysis 1

In the main manuscript, we compared the performance of the proposed classifier which used combination of features from CAT12 and MRIQC against the default versions of existing tools (CAT12 and MRIQC). Here, we present results from an additional supplementary analysis, where we applied the RUS classifier (the best-performing classifier on the combined dataset) in two further scenarios: 1) using only MRIQC features, and 2) using only CAT12 features. The aim of this supplementary analysis was to determine if indeed the combination of features improves the RUS model's performance.

To do this, we fully re-trained our RUS model with combined features and saved the random generator seeds for each of the cross-validation (CV) iterations. These seeds were then used to ensure that the same samples are selected during training and test data splits in the cross-validation iterations. This approach allowed fair comparison of classification performances across 100 runs of CV between the three approaches. The classification design framework was exactly similar to the one described in section 2.5 of the main paper except the number and types of feature sets (CAT12 – 36 features, MRIQC – 68 features, combined – 104 features). The classifiers were trained on different feature sizes for e.g., for CAT12 – 10,20,30,36, MRIQC – 10,20,30,40,50,60,68 and combined – 10,20,30,40,50,60,70,80,90,100,104. The trained classifiers were tested on the hold-out test data and balanced accuracies were compared mainly for model with only CAT12 (36) features, model with MRIQC (68) features and combined model with 80 features which is also the reported feature size in the main paper. We observed that the RUS classifier with combined features showed the highest balanced accuracy of 88.4% as compared to only using MRIQC features (82.4%) and CAT12 features (85.2%) on the test dataset. It is also interesting to note that the performance of MRIQC-features-only and CAT12-features-only classifiers designed using our RUS approach was higher than that of the default tools (MRIQC-default – 71.6%, CAT12-default – 56.9%).

Given the small test set size, we conducted additional statistical analyses for model performance on 100 cross-validation runs and the final test data. This allowed us to assess model stability and variability across different feature sizes and models (CAT12-only, MRIQC-only, combined features).

Model variability – Across 100 runs of cross validation

1) Balanced accuracy across CV runs and feature sizes

First, we visualised balanced accuracy across 100 cross validation runs to show how model performance varies with feature sizes (***Figure S6***). The combined features model consistently had overall higher median balanced accuracy and less variability compared to both the CAT12-only and MRIQC-only models. Notably, performance of combined model was stable after 80 feature size (the reported feature size for the combined model).

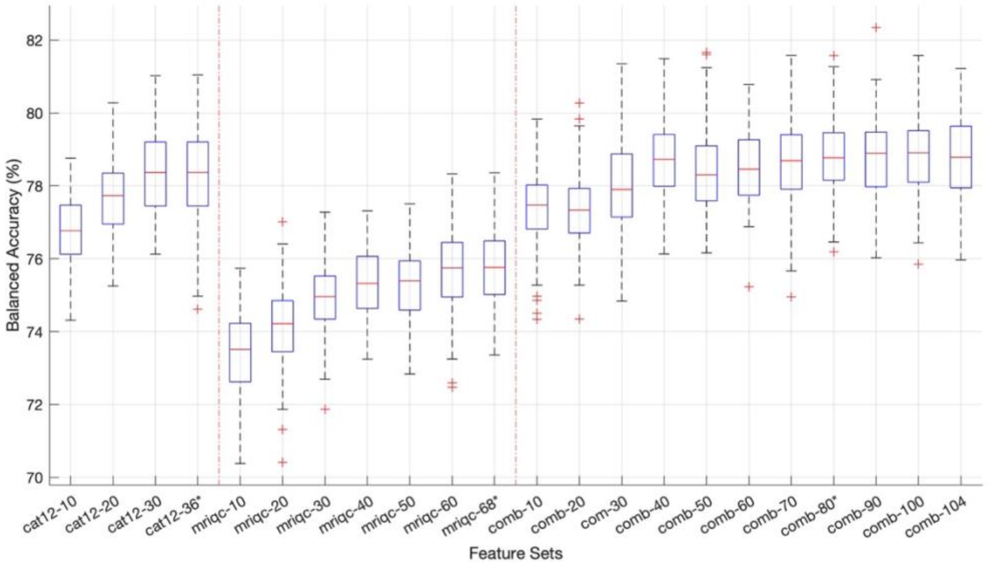

Figure S6. Balanced accuracies across 100 cross validation runs for each feature size. The * shows the feature size at which the performance is reported on unseen test data. The red vertical lines distinguish three approaches – using only CAT12 features, only MRIQC features and combination of both.

2) Bootstrapping analysis

To further evaluate the stability and generalisability of the models, we conducted bootstrapping with 10,000 iterations to estimate the 95% confidence intervals (CI) and standard deviations (Std CI) for each model and feature size (***Figure S7***). The results confirmed the combined approach's superior performance with the lowest variability. Specifically, for the CAT12-only features, performance improves as the number of features increases, with all features (36) showing a balanced performance (mean CI = 78.07 – 78.55), though with a slight increase in variability (std CI = 0.12). In the case of MRIQC-only features, the performance exhibits an improvement across feature sizes, with all features (68) achieving mean CI of 75.59 – 75.99 (std CI = 0.10). The combined model demonstrates a consistent improvement and stable performance after 80 feature size – providing the mean CI (78.59 – 79.00) and the lowest standard deviation of confidence intervals (std CI = 0.11). Even though the variability is similar

across three approaches, the combined approach outperforms the two in terms of higher balanced accuracy.

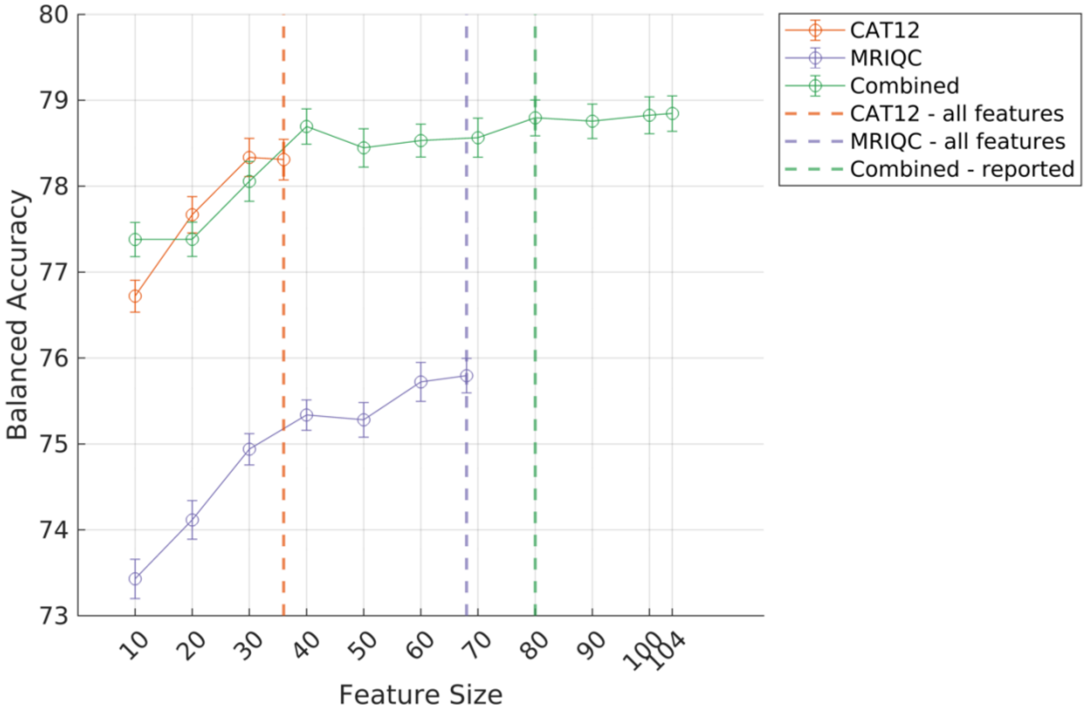

Figure S7.Bootstrapping analysis of model performance across 100 cross-validation runs. The figure shows the 95% confidence intervals (CI) for the mean balanced accuracy, calculated over 10,000 bootstrap iterations.

3) Statistical analysis

Additionally, we statistically compared balanced accuracies across 100 CV runs specifically for feature sizes – combined model with 80 features, model with only CAT12 features (total 36), model with only MRIQC features (total 68). Given that the data was not normally distributed, as confirmed by the Kolmogorov-Smirnov, we performed non-parametric tests to compare the balanced accuracies. A Friedman test was conducted to evaluate differences in balanced accuracies across three models. The test revealed a statistically significant difference in performance, $\chi^2(2)=133.44$, $p<0.001$. The mean ranks were 2.28 for CAT12 models, 1.08 for MRIQC models, and 2.64 for combined models, indicating that the model with combined features had the highest balanced accuracies, followed by CAT12, and MRIQC features being the lowest. Pairwise Wilcoxon signed-rank tests further confirmed significant differences between combined model and MRIQC-only model ($p<0.001$, $z=8.7$, signed rank=5047) and between combined features and CAT12-only model ($p<0.005$, $z=3.0$, signed rank=3401). These results suggest that the combination of features leads to significantly

higher balanced accuracies for different sets of training and test folds in cross validation as compared to using the features from the individual tools.

Model variability – performance on the test data

1) Performance across different feature sizes

We visualised model performance on the test data across different feature sizes (***Figure S8***). The combined features model consistently outperformed the others at most feature sizes, with stable performance after 80 features. The CAT12-only model showed improvement with increasing feature size but remained lower than the combined model, while the MRIQC-only model exhibited high fluctuations in performance

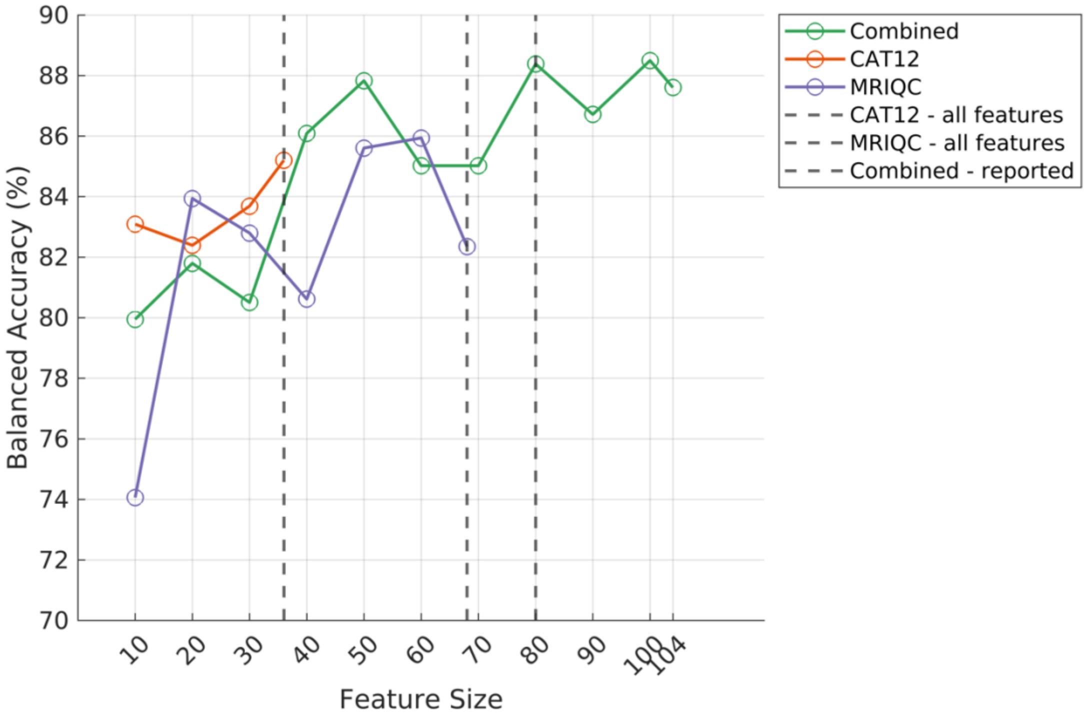

Figure S8. Comparison of RUS classifier performances across different feature sizes for models trained and tested using only CAT12 features, only MRIQC features and combination of both. The proposed RUS model (with combined features) was fully re-trained with saved random generator seeds, which were then used to fully train models using only MRIQC and CAT12 features and finally balanced accuracies were compared.

2) Bootstrapping analysis using predicted and true labels

To further assess the uncertainty in the performance estimates of our models (combined model with 80 features, model with 36 CAT12 features and model with 68 MRIQC features) and considering the smaller test set in our study, we used bootstrapping procedure. We estimated the 95% confidence interval (CI) around the balanced accuracy by resampling pairs of true and predicted labels with replacement for 10,000 iterations. The model with combined features yielded the highest mean balanced accuracy of 88.4% (95% CI: 82.2 – 93.7%), while that with CAT12-only model and MRIQC-only model had mean balanced accuracies of 85.2% (95% CI: 78.4 – 91.2%) and 82.4% (95% CI: 75.1 – 88.8%), respectively. To evaluate the significance of performance differences in pairwise manner, we then conducted Wilcoxon signed-rank tests. The results showed statistically significant differences between all pairs of classifiers, with p-values <0.001 for both the combined vs. CAT12-only features (*z*-value=78.5) and combined vs. MRIQC-only (*z*-value=86.6) comparisons. These results indicate substantial performance disparities among the classifiers and suggest that the combined model significantly outperforms the other two approaches.

3) Comparing classification error rates

To assess whether the observed differences in error rates between the models could be attributed to sampling noise or represent a true performance distinction, we used McNemar's test to compare the three models – combined model with 80 features, CAT12-only with 36 features, and MRIQC-only with 68 features. The combined model achieved the lowest misclassification rate (11.2%) as compared to CAT2-only (14.5%) and MRIQC-only (17.2%) models. McNemar's test revealed that the combined model significantly outperforms both the CAT12-only model (p<0.05) and the MRIQC-only model (p<0.0001) in terms of error patterns. These results suggest that the combined model has a significantly lower error rate compared to the other models, demonstrating its better overall performance.

In summary, our extensive evaluation of model variability on performance across 100 cross-validation runs and final test data confirms that the combined model consistently outperforms the models designed with either CAT12 or MRIQC features. The combined model not only achieves significantly higher balanced accuracy but also demonstrates greater reliability, and lower variability. Our statistical analyses (bootstrapping, Friedman test, Wilcoxon test, and McNemar test) confirm that the differences observed between these models are statistically significant and underscore the superiority of the combined approach. Please note that the

codes and data that were used to perform these analyses are now available on our GitLab repository.

Supplementary analysis 2

The primary emphasis of the paper is on our final model – **combined data model**. Our intention in the main paper was to explore how well the hyperparameters and feature rankings derived from the combined data model could generalise to new, unseen datasets, similar to transfer learning where previously learned knowledge is reused. The leave-one-site-out and exploratory models presented in the paper serve primarily to demonstrate the robustness of our training approach across various configurations of training and testing datasets. They are (partially) trained on different datasets re-using the hyperparameters and features that were ranked according to the final model (combined data model), hence some data leakage is expected.

In this analysis, we provide the results when the leave-one-site out and exploratory models (on field strength and manufacturers) are fully trained (i.e. optimising the hyperparameters of the RUS classifier and feature ranking within the nested cross validation approach). ***Figure S9*** shows the workflow of leave-one-site out models trained from scratch (meaning fully trained). The workflow to fully retrain the exploratory model is the same as the one used to train the main model (*Figure 1* in the main text). The other components of the training workflow such as feature preprocessing, feature ranking methods, feature sizes, classifiers and optimisation strategies were same as described in the main paper (*Refer Section 2.5.1 Combined data model*). The performances of these models (balanced accuracy) are provided below across each feature size (Refer ***Figure S10*** for leave-one-site out, ***Figure S12*** for exploratory models). Also, we provide the plots with mean and standard deviation across all the feature sizes comparing both the approaches i.e. partially trained models (presented in paper) vs. fully trained models (Refer ***Figure S11*** for leave-one-site out and ***Figure S13*** for exploratory models).

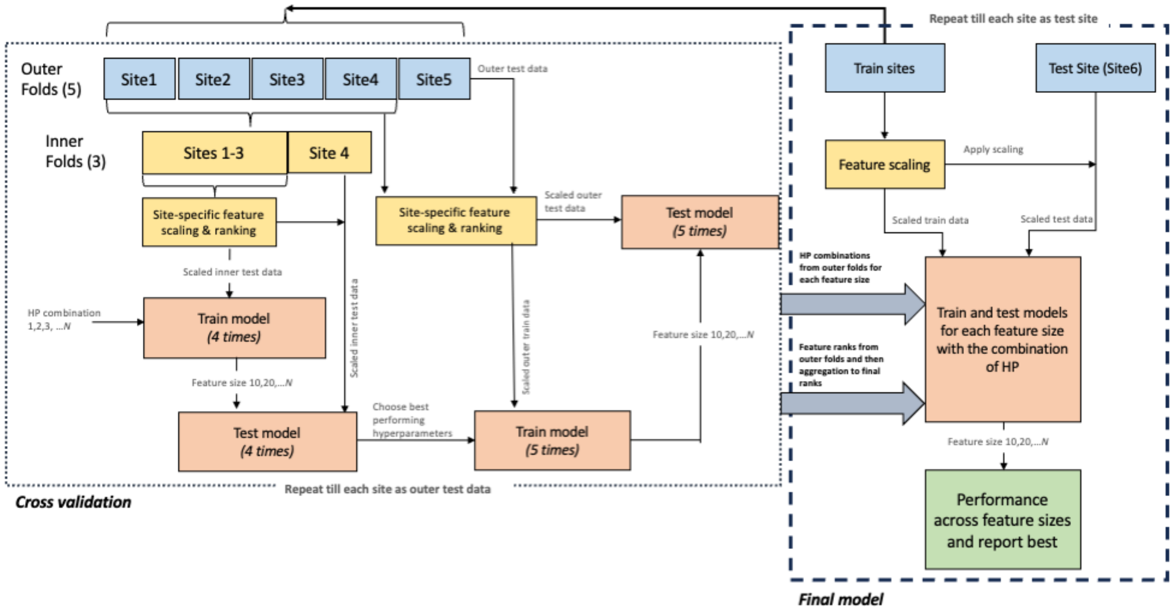

Figure S9. Cross validation workflow for training leave-one-site out models from scratch (fully trained). The models were trained on data from remaining sites while keeping each site as test data. The hyperparameters of the model were optimised on the inner test data and the combination giving the best performance were selected for the outer folds. The process was repeated till each site was used as test data. The best performing hyperparameters for each feature size and feature ranks across outer cross validation iterations were used to train the final model and tested on the test site.

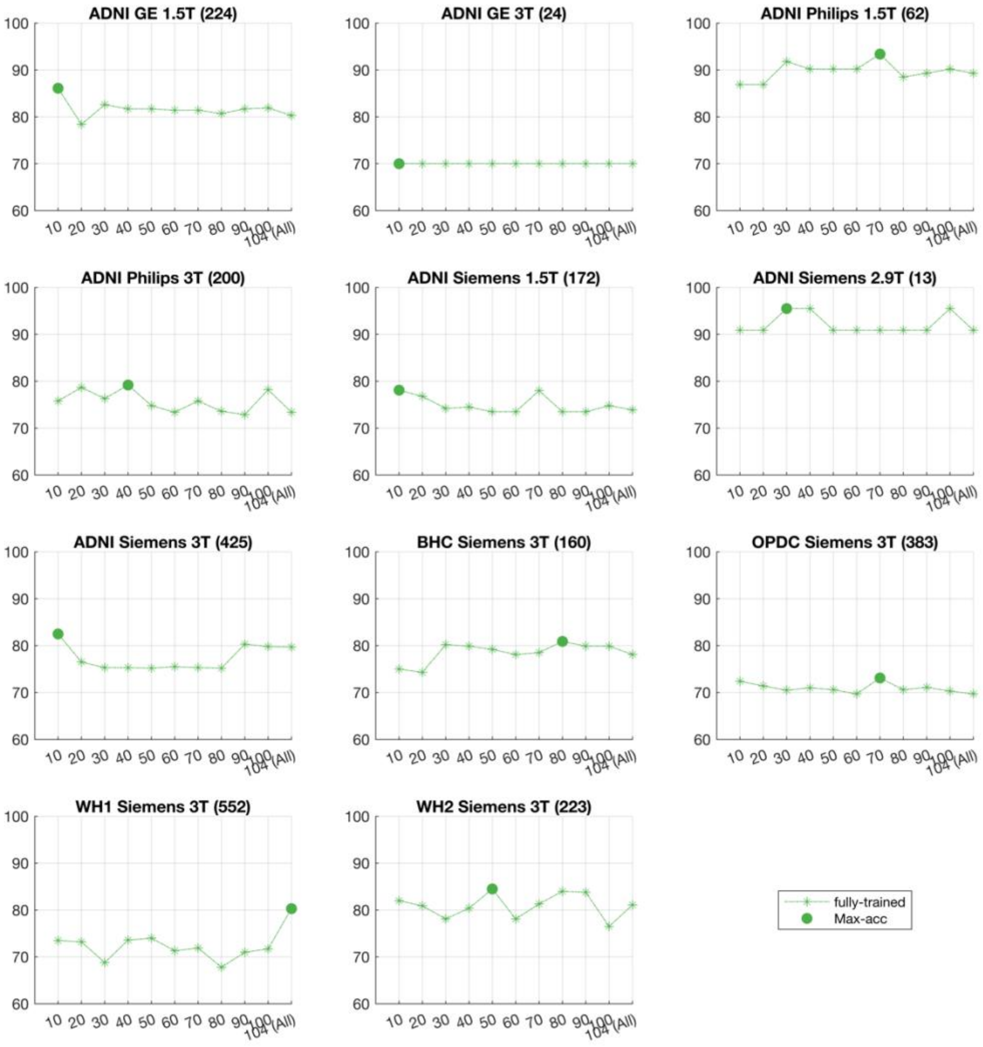

Figure S10. Balanced accuracy of proposed RUS classifier across different feature sizes for 'fully trained' leave-one-site-out models. The maximum balanced accuracy across all the feature sizes for each site are highlighted.

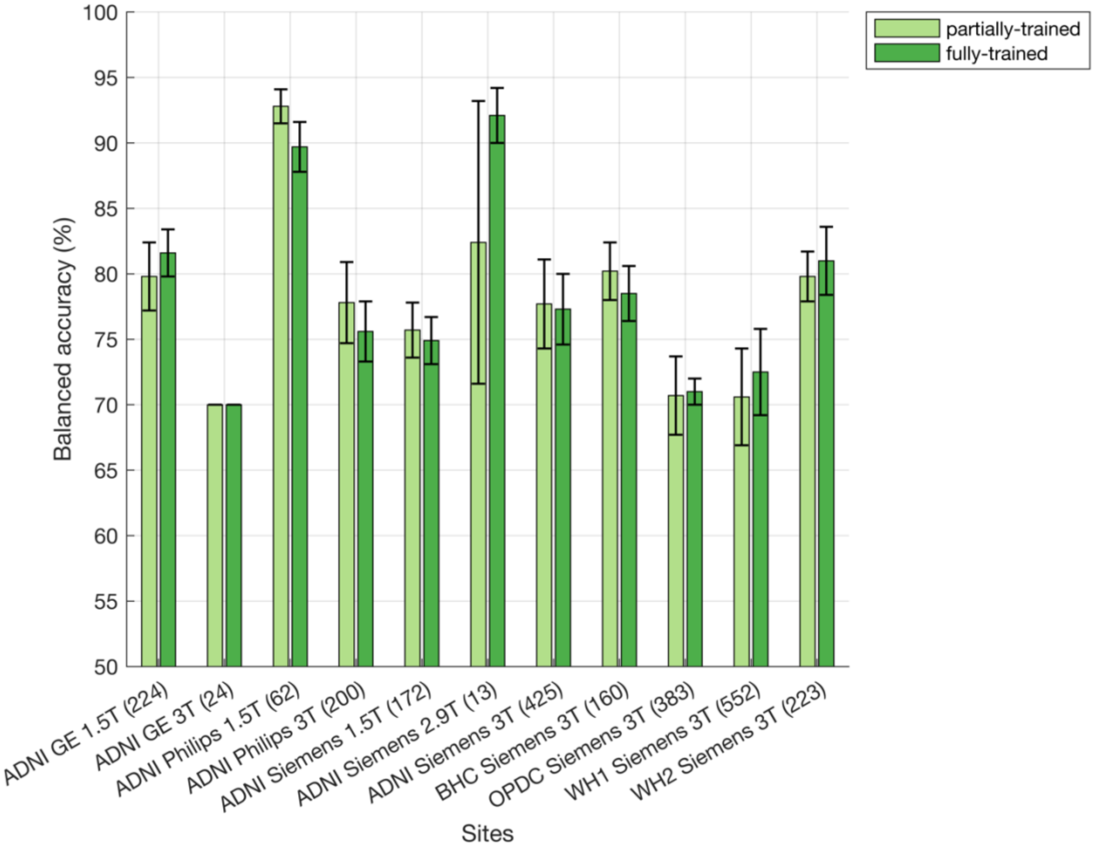

Figure S11. Mean and standard deviation of balanced accuracies across feature sizes comparing the two approaches partially trained versus fully trained leave one site out models

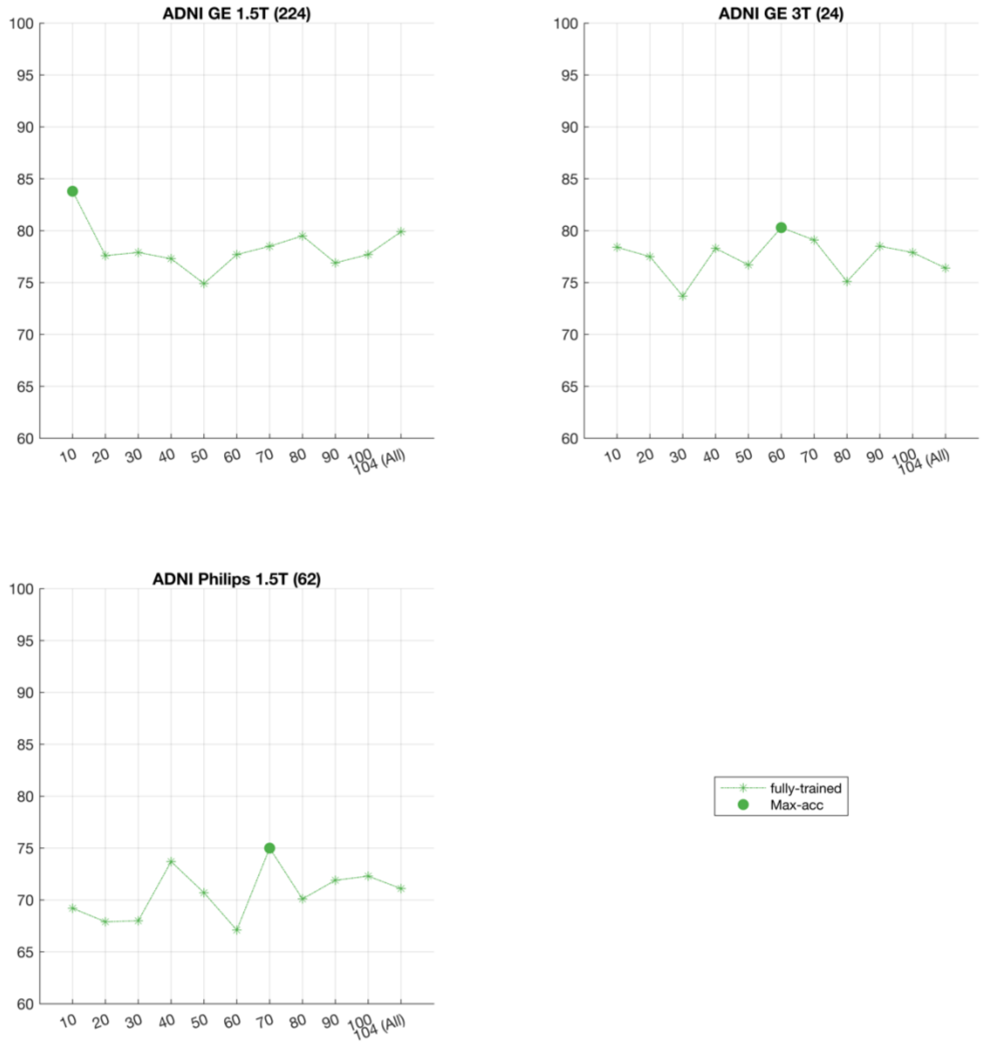

Figure S12. Balanced accuracy of proposed RUS classifier across different feature sizes for 'fully trained' exploratory models. The maximum balanced accuracy across all the feature sizes is highlighted.

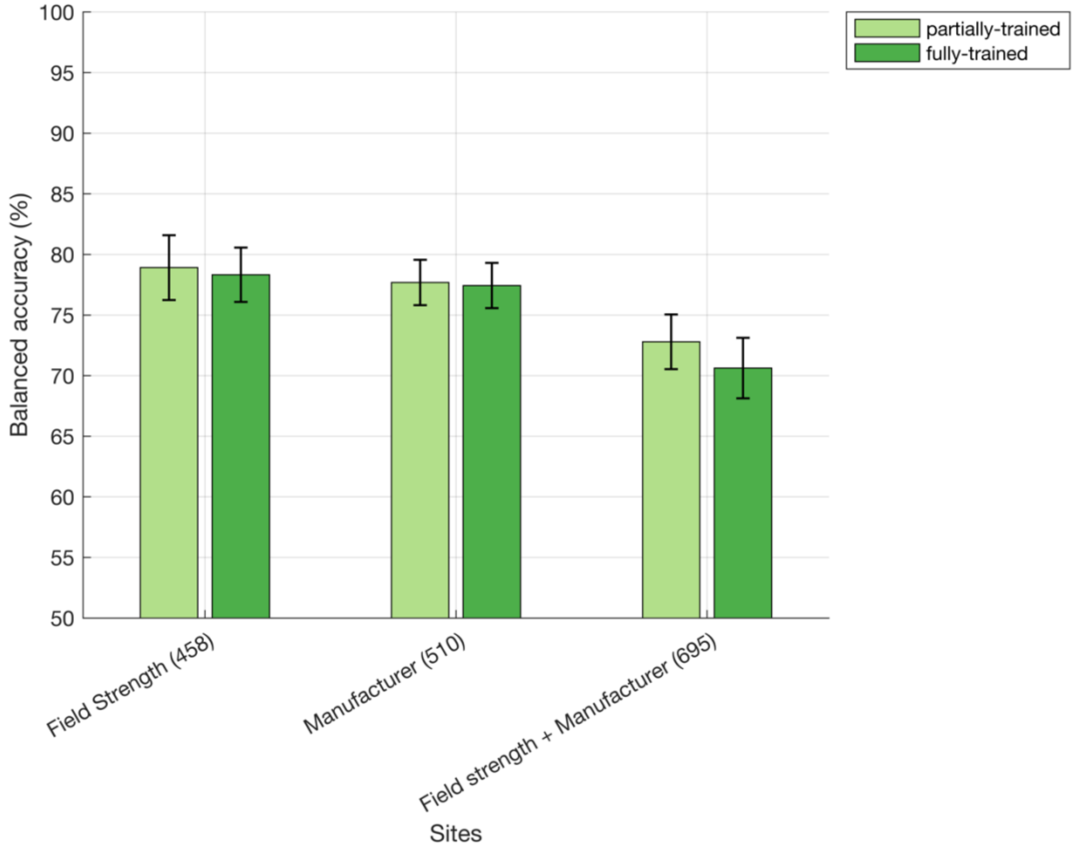

Figure S13. Mean and standard deviation of balanced accuracies across feature sizes comparing the two approaches partially trained versus fully trained exploratory models

Supplementary analysis 3

Despite including a wide variety of datasets on ageing and neurodegeneration, we acknowledge that one of the limitations of our study is that the total number of samples in the reject class is low (total samples in test dataset 483, reject 33) which might impact the generalisability of our classifier to wider datasets. While we discussed this limitation in the main paper, to test whether the proposed classifier could be used also on a very different population to those originally trained with, in terms of both demographics and level of artefacts, we used Movement-Related ARTefacts (MR-ART) dataset. Briefly, this dataset was designed to include both motion-free (N=148) and motion-affected data (N=288) acquired from the same young healthy participants (age mean age 30 ± 13 years). We tested the combined data model directly on the MR-ART dataset, as well as the option of incorporating samples from MR-ART in the training data (between 1% and 90%). While our classifier is designed on ageing and neurodegenerative populations, we can still see the value of testing our proposed classifier on MR-ART, to answer a question: can our classifier generalise to scans from healthy young individuals, outperforming existing QC algorithms also in that context? If not, can the performance be improved by incorporating training examples from a different population to the ones for which the classifier was originally designed? To this aim, we performed the analyses described below.

The T1w images from the MR-ART dataset were processed in MRIQC and CAT12 to extract the image quality metrics. For ground truth generation, *scores.tsv* provided by dataset authors was used. We generated binary labels by binning scans with scores 1 (good) and 2 (medium) into accept category and 3 (bad) into reject category. See ***Table S2*** for the counts of accept and reject scans for two acquisition labels in this dataset – standard (no head motion in the scanner) and head motion (slight or excess head motion in the scanner). Our trained classifier (combined data model) was used to predict QC. The performance of our classifier was compared with the ground truth and with default versions of other tools – CAT12 ratings (weighted IQR below C minus as reject) and MRIQC classifier predictions (scans labelled as 1 by the MRIQC classifier as reject). The results are shown in **Figure S*14***.

Table S2. Visual QC counts for MR-ART dataset

Acquisition labels	Total	Accept	Reject
Standard	148	147	1
Head motion	288	91	197
All	436	238	198

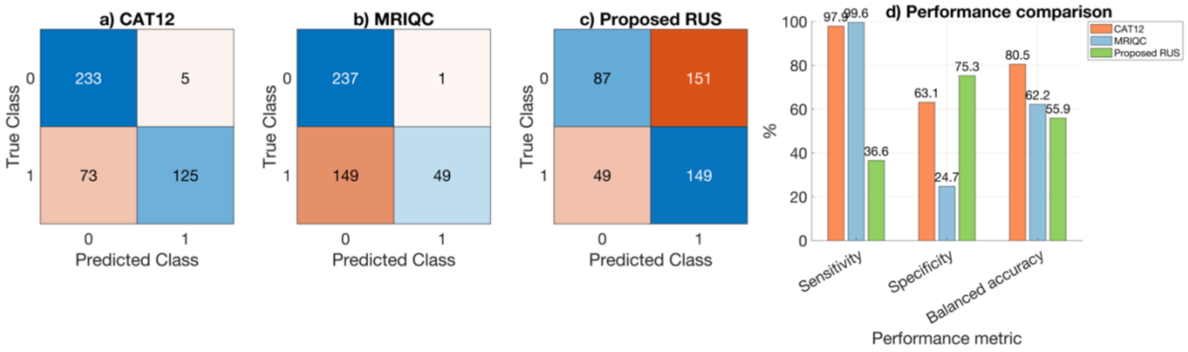

Figure S14. Confusion matrices (panels a-c) and performance comparing the CAT12 ratings, MRIQC classifier and proposed (RUS) classifier (panel d) on data from the MR-ART dataset. In confusion matrices, 0 is for accept class and 1 is for reject class.

Our RUS classifier showed lower balanced accuracy than MRIQC and CAT12. While RUS demonstrated lower sensitivity (ability to correctly predict accept-quality scans) than both CAT12 and MRIQC, it outperformed them in specificity (ability to correctly predict reject-quality scans). Notably, RUS correctly identified more reject-quality scans as compared to CAT12 and MRIQC (***Figure S14***, panel a – CAT12: 125/197, panel b – MRIQC: 49/197, panels c – RUS: 149/198). However, it struggled predicting the accept class, correctly predicting only 87/238 scans (***Figure S14*,** panel c).

While CAT12 demonstrated the highest performance on the MR-ART dataset, it is important to note that its performance was the lowest on the test dataset used in our main analyses (balanced accuracy: CAT12 – 56.9%, MRIQC – 71.6%, RUS – 87.7%). This suggests that while CAT12 is well suited to datasets from healthy young individuals like MR-ART, its ability to handle datasets from ageing and neurodegenerative populations, such as those used in our main study might be limited. Similarly, as shown in ***Figure S14*** panel c, out of the three QC methods, MRIQC misclassified the highest number of reject-quality scans as accept (MR-ART: 148/197; test dataset in the main analyses: 17/33) compared to our classifier (MR-ART: 49/197; our test dataset in the main analysis: 4/33). These results show that the RUS classifier is more robust towards predicting reject quality scans at the expense of flagging more accept quality scans into the reject class. Importantly, our classifier's conservative approach—erring on the side of caution—ensures that fewer reject-quality scans are mistakenly retained for further analysis (criteria similar to UK biobank pipeline's autoQC (Alfaro-Almagro et al., 2018)). This characteristic is crucial for large-scale neuroimaging studies, where incorrectly approving poor-quality scans can be more detrimental than rejecting acceptable-quality scans.

We think that several factors likely contributed to the performance on the lower side when validating RUS classifier on datasets such as MR-ART:

- Differences in Study Populations – As previously mentioned, the MR-ART dataset consists of scans from a younger, healthy population (mean age 30 ± 13 years), whereas our dataset primarily includes elderly participants (mean age = 71 ± 8 years). Since RUS was optimised for age-related structural variations, its performance may not generalise well to younger populations.
- Ground Truth labelling – While we trained our classifier on visual QC ratings from different datasets used in this study, the visual ratings followed similar criteria. However, it is possible that small differences in the visual quality rating criteria and thresholds across datasets may influence the classifier's performances. In the MR-ART dataset, bad quality images were those considered unusable for clinical diagnostics, which is a potentially more lenient threshold than what used for a research scan (i.e. an image could still be reported visually but be too corrupted to generate reliable measures when analysed with automated tools). This could contribute to explain why our classifier rated more MR-ART scans in the reject class.
- Variations in Defacing Methods – The MR-ART dataset was shared using pydeface, whereas our dataset was defaced using fsl_deface. Prior studies (Bhalerao et al., 2022; Provins et al., 2023; Rubbert et al., 2022) indicate that defacing methods can significantly impact quality metrics, potentially affecting classifier performance.

We then explored whether introducing a subset of labelled samples (with ground truth) from this new dataset could enhance the classifier's ability to generalise. The rationale behind this approach is that exposure to dataset-specific patterns may enable the classifier to adapt more effectively, potentially improving its robustness across diverse datasets.

To investigate this, we applied a retraining procedure similar to the methods outlined in Section 2.5.2 of the main paper ('Additional validation of combined data model'). This follows a training approach like transfer learning, where we use parameters from the trained model (combined data model) —including feature ranking, feature size and hyperparameters—while augmenting the training set with a subset of MR-ART samples. The updated training dataset thus consisted of our original training dataset (N = 1955) supplemented with additional labelled MR-ART samples. Importantly, this retraining process was computationally efficient, as it did not involve hyperparameter re-optimisation but simply expanded training set. By incorporating samples from MR-ART in the training data, we aimed to – a) assess whether our classifier's performance is improved 2) determine the minimum number of additional samples required to achieve a more balanced performance (improved sensitivity and specificity). More specifically, the dataset was split into training and testing by holding out samples from MR-ART in a

stratified manner (*cvpartition* in *MATLAB R2023a*). The training samples were added to the original training data for re-training (***Table S3***).

Table S3. Training and testing split for exploratory models on the MR-ART dataset.

Models	Training datasets	N training – Total (accept)	MR-ART N training – Total (accept)	MR-ART N test – Total (accept)
Combined data model	All main datasets (table 4)	1955 (1815)	0	436 (238)
1% MR-ART	All main datasets (table 4) and 1% MR-ART samples	1961(1819)	6(4)	430(234)
5% MR-ART	All main datasets (table 4) and 5% MR-ART samples	1979(1829)	24(14)	412(224)
30% MR-ART	All main datasets (table 4) and 30% MR-ART samples	2088(1888)	133(73)	303(165)
60% MR-ART	All main datasets (table 4) and 60% MR-ART samples	2131(1911)	263(144)	173(94)
80% MR-ART	All main datasets (table 4) and 80% MR-ART samples	2305(2006)	350(191)	86(47)
90% MR-ART	All main datasets (table 4) and 90% MR-ART samples	2349(2031)	394(216)	42(22)

Figure S15 and ***Table S4*** illustrates the impact of incorporating labelled MR-ART samples into the training process on the classifier's performance. The results indicate that RUS re-trained by adding subset of MR-ART samples to the original training dataset ('RUS with MR-ART training' – green line) consistently outperforms MRIQC and CAT12 tools (balanced accuracy, >87%). Notably, specificity remains significantly higher for RUS (>92%) than before and sensitivity is also substantially improved (>78%) as compared to previous performances. While MRIQC & CAT12 achieves relatively higher sensitivity (MRIQC >99%, CAT12 >98%), it is at the cost of low specificity (MRIQC <26%, CAT12 <65%), leading to a higher risk of incorrectly predicting bad-quality scans to accept. Importantly, even as few as adding 6 samples from MR-ART contribute to substantial boost in performance, reinforcing the effectiveness of this domain adaptation approach (balanced accuracy – improved from 55.6% to 88.5%, sensitivity – improved from 36.5% to 84.6%, specificity – improved from 75.3% to 92.3%).

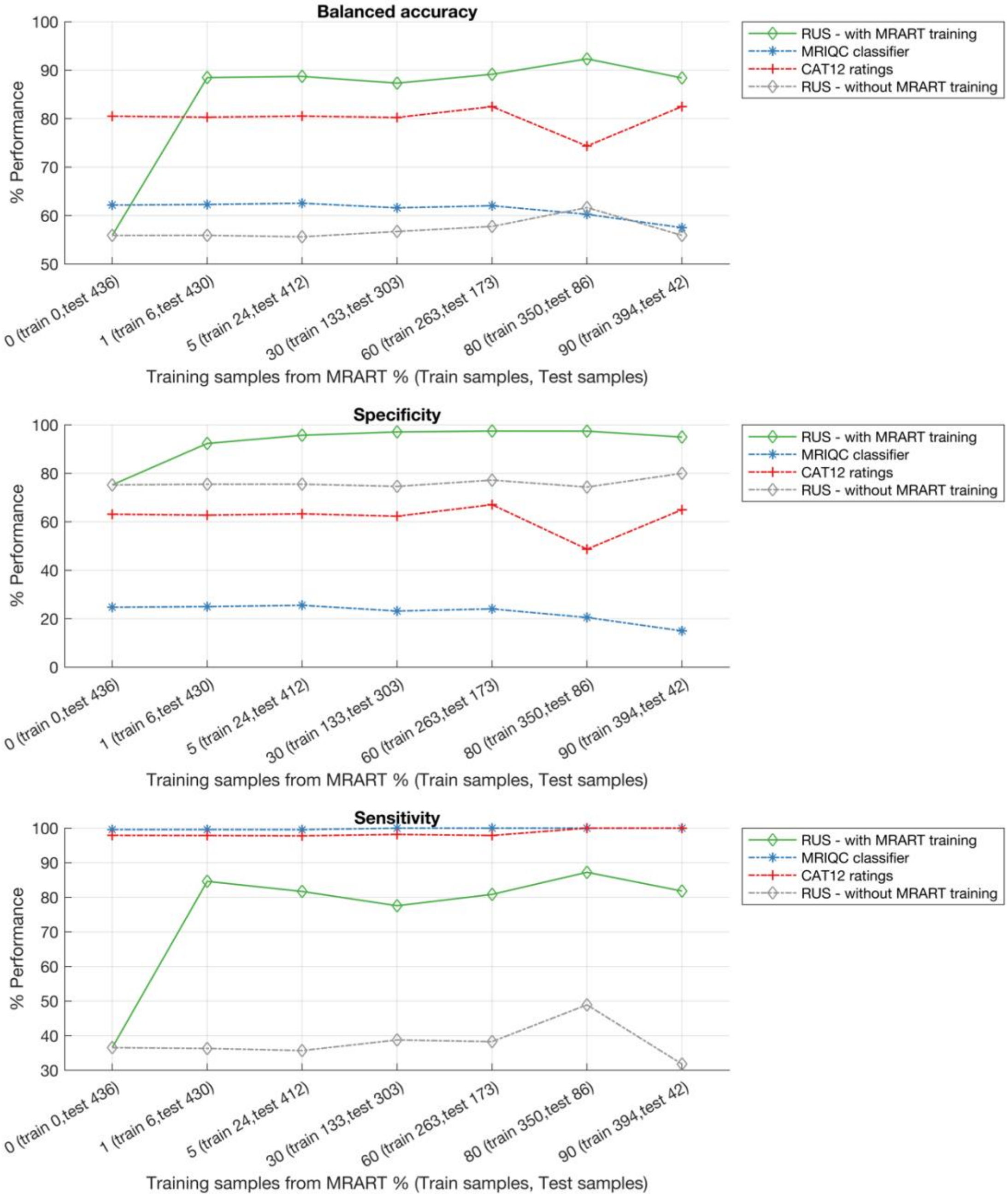

Figure S15. Balanced accuracy, specificity and sensitivity (y-axis) with (‘RUS- with MR-ART training) and without adding MR-ART samples (RUS – without MR-ART training) to the original training dataset for re-training RUS model. X-axis indicates the % of MR-ART data added to the original training data and total number of training and test samples from MR-ART.

Table S4. Balanced accuracy, specificity and sensitivity on subsets of MR-ART test data (column 1) excluding a proportion of samples that are added to the main training dataset for re-training. The performances are provided for the combined data model (column 2, without MR-ART in training, N = 1955), default CAT12 (column 3), default MRIQC (column 4) and re-trained RUS (column 6) with proportion of MR-ART samples added to the main training data (column 5).

MR-ART Test data	Combined data model (RUS) (*N* = 1955 training)	Default CAT12	Default MRIQC	MR-ART Train data	Re-trained RUS [All main datasets *N* = 1955 + MR-ART samples in previous column]
MR-ART N Total(accept)	*Balanced accuracy (%)*	*Balanced accuracy (%)*	*Balanced accuracy (%)*	*MR-ART N Total(accept)*	*Balanced accuracy (%)*
436 (238)	55.9	80.5	62.2	0	55.9
430(234)	55.9	80.3	62.3	6(4)	88.5
412(224)	55.6	80.5	62.5	24(14)	88.7
303(165)	56.7	80.3	61.6	133(73)	87.3
173(94)	57.8	82.5	62.0	263(144)	89.2
86(47)	61.6	74.4	60.3	350(191)	92.3
42(22)	55.9	82.5	57.5	394(216)	88.4
Test data	Combined data model (RUS) (*N* = 1955 training)	Default CAT12	Default MRIQC	Train data	Re-trained RUS [All main datasets *N* = 1955 + MR-ART samples in previous column]
MR-ART N Total(accept)	*Specificity (%)*	*Specificity (%)*	*Specificity (%)*	*MR-ART N Total(accept)*	*Specificity (%)*
436 (238)	75.3	63.1	24.7	0	75.3
430(234)	75.5	62.8	25.0	6(4)	92.3
412(224)	75.5	63.3	25.5	24(14)	95.7
303(165)	74.6	62.3	23.2	133(73)	97.1
173(94)	77.2	67.1	24.1	263(144)	97.5
86(47)	74.4	48.7	20.5	350(191)	97.4
42(22)	80.0	65.0	15.0	394(216)	95.0
Test data	Combined data model (RUS) (*N* = 1955 training)	Default CAT12	Default MRIQC	Train data	Re-trained RUS [All main datasets *N* = 1955 + MR-ART samples in previous column]
MR-ART N Total(accept)	*Sensitivity (%)*	*Sensitivity (%)*	*Sensitivity (%)*	*MR-ART N Total(accept)*	*Sensitivity (%)*
436 (238)	36.6	97.9	99.6	0	36.6

430(234)	36.3	97.9	99.6	6(4)	84.6
412(224)	35.7	97.8	99.6	24(14)	81.7
303(165)	38.8	98.2	100.0	133(73)	77.6
173(94)	38.3	97.9	100.0	263(144)	80.9
86(47)	48.9	100.0	100.0	350(191)	87.2
42(22)	31.8	100.0	100.0	394(216)	81.8

These findings underscore the benefit of incorporating domain-specific training data to improve the generalisability of QC classifiers across diverse datasets. While CAT12 ratings demonstrate strong sensitivity, their lower overall performance on other datasets raises concerns about robustness. MRIQC provides good sensitivity but lacks the specificity necessary for stringent QC applications. The proposed RUS classifier, particularly after augmenting training data, boosts specificity as well as sensitivity, thereby balancing both metrics effectively. The observation that only a handful of samples (e.g., 6 from MR-ART) significantly enhanced classifier performance suggests that such a strategy can be valuable when adapting our QC model to new datasets. This suggests that our classifier benefits from lightweight domain adaptation without requiring full hyperparameters re-optimisation. Future studies could explore strategies for optimal sample selection to ensure adaptability across diverse neuroimaging datasets.
